# Physician Variation in Early Sepsis Management

**DOI:** 10.1001/jamanetworkopen.2025.56945

**Published:** 2026-02-13

**Authors:** Ithan D. Peltan, Danielle Groat, Jorie Butler, Joseph R. Bledsoe, Blessing S. Ofori-Atta, Chaorong Wu, Angela P. Presson, Tom H. Greene, Matthew A. Christensen, Fiona M. Schroeder, M. Blakely May, Matthew H. Samore, Catherine L. Hough, Samuel M. Brown

**Affiliations:** 1 Department of Pulmonary and Critical Care Medicine, Intermountain Medical Center, Salt Lake City, Utah; 2 Division of Geriatrics, Department of Internal Medicine, University of Utah School of Medicine, Salt Lake City; 3 Department of Emergency Medicine, Intermountain Medical Center, Salt Lake City, Utah; 4 Department of Population Health Sciences, University of Utah School of Medicine, Salt Lake City; 5 Division of Epidemiology, Department of Medicine, University of Utah School of Medicine, Salt Lake City; 6 Division of Allergy, Pulmonary and Critical Care Medicine, Department of Medicine, Vanderbilt University Medical Center, Nashville, Tennessee; 7 Office of Consumer Experience, Intermountain Health, Salt Lake City, Utah; 8 Division of Pulmonary and Critical Care Medicine, Department of Medicine, Oregon Health & Sciences University, Portland; 9 Division of Pulmonary and Critical Care Medicine, Department of Medicine, University of Utah School of Medicine, Salt Lake City

## Abstract

**Importance:**

Prompt antimicrobial therapy is essential in sepsis, but accelerating antimicrobial administration may increase overtreatment.

**Objectives:**

To examine the extent of and factors associated with physician variation in time from emergency department (ED) presentation to antimicrobial administration (hereinafter termed *door-to-antimicrobial time*) for sepsis and to assess whether faster practice patterns are associated with overtreatment.

**Design, Setting, and Participants:**

This explanatory mixed-methods study linked a quantitative retrospective cohort (July 1, 2013, to January 31, 2017) involving 30-day patient follow-up with prospective qualitative physician interview data (May 17, 2022, to June 28, 2023) at 4 Utah EDs. Participants included ED attending physicians and their patients meeting sepsis criteria (including intravenous antimicrobial administration) before ED departure. Data analysis occurred from 2021 to 2025.

**Main Outcomes and Measures:**

Assessment for physician door-to-antimicrobial time variation used a likelihood ratio test comparing a linear mixed-effects model incorporating physician-level random intercepts and patient-level covariates with a model without physician random effects. Empirical best linear unbiased predictions of the physician random intercepts (termed *physician-predicted mean door-to-antimicrobial times*) quantified variation. The primary analysis used a joint mixed-effects shared parameter model to evaluate the association of physicians’ door-to-antimicrobial practice patterns with their overtreatment rate (infection ruled out on final retrospective adjudication). Qualitative analysis of semistructured cognitive task analysis interviews compared ED physicians in the fastest and slowest door-to-antimicrobial time quartiles.

**Results:**

Quantitative analyses included 88 ED physicians (71 [80.7%] male; median age, 39 [IQR, 35-49] years) and 9810 patients with sepsis (median age, 63 [IQR, 48-75] years), of whom 4635 (50.5%) were female and 3540 (38.6%) received antimicrobials more than 3 hours after ED arrival. The median number of patient encounters per physician was 105 (IQR, 75-129). Physicians’ door-to-antimicrobial time varied significantly (likehood ratio test *P* < .001), with average physician-level estimated mean door-to-antimicrobial time of 184 (95% estimation interval, 146-222) minutes for a typical patient, but was not associated with overtreatment (adjusted odds ratio, 0.98 [95% CI 0.94-1.02] per 10-minute increase in physician estimated mean door-to-antimicrobial time; *P* = .37). Among 18 physicians interviewed, physicians with faster door-to-antimicrobial times emphasized proactive, parallel task execution and care team coordination, while physicians with slower times described a more reactive and stepwise sepsis evaluation and treatment process.

**Conclusions and Relevance:**

In this mixed-methods study, ED physicians’ antimicrobial administration time for sepsis varied significantly, but faster antimicrobial initiation practice patterns were not associated with overtreatment. Physicians with shorter door-to-antimicrobial times described a proactive, parallel processing approach to sepsis care.

## Key Points

**Question:**

Are sepsis treatment practice patterns characterized by faster antimicrobial initiation associated with increased overtreatment, and what are their mechanisms?

**Findings:**

In this mixed-methods study of 9810 patients and 88 treating physicians, emergency department physicians with faster patterns of antimicrobial initiation practice described a proactive, parallel processing approach to sepsis care and empowerment to overcome system-level obstacles but did not exhibit increased overtreatment.

**Meaning:**

These findings suggest that individual and team-based methods to facilitate prompt antimicrobial administration for sepsis are unlikely to increase unnecessary antimicrobial treatment.

## Teaser

This mixed-methods study assesses the patterns of emergency department physicians who treat patients with suspected sepsis and the association of shorter time to antimicrobial administration with overtreatment.

## Introduction

Early, appropriate antimicrobial therapy is essential for patients with sepsis,^1,2,3,4,5,6,7^ the common and deadly syndrome resulting when a dysregulated immune response to infection causes acute organ failure.^8,9,10^ Nevertheless, prompt delivery of high-quality sepsis care remains challenging, with many patients receiving antimicrobials after the recommended 3-hour window.^2,3,4,7,11^

Decision aids and care reorganization can help identify patients with potential sepsis, facilitate diagnostic evaluation, and expedite treatment initiation.^4,12,13,14^ Such measures, however, may result in adverse spillover effects, including antimicrobial overtreatment.^14,15,16,17,18,19^ At the same time, numerous patient and system factors unrelated to patient preferences, complexity, and illness severity appear to influence antimicrobial timing for community-acquired sepsis.^20,21,22,23,24^ A pilot study including investigators from our team^25^ provided preliminary evidence of physician-level variation for time from patient presentation to antimicrobial administration (hereinafter termed *door-to-antimicrobial time*), with physicians’ practices differentially influenced by infection manifestations and illness severity. Together, these findings suggest a need to better understand how and why care organization and physician decision making influence sepsis evaluation and treatment.

Analyses combining quantitative and qualitative methods can aid robust characterization of the mechanisms underlying practice variation and guide measures to overcome them. We therefore performed a mixed-methods study to investigate the extent and drivers of physician variation for timing of antimicrobial administration in sepsis and exploit observed practice variation to measure potential spillover from accelerated antimicrobial treatment.

## Methods

### Study Design

This explanatory mixed-methods study—a design in which quantitative results inform qualitative inquiry designed to explain the quantitative findings—linked quantitative analyses investigating physician treatment behaviors in a retrospective cohort of ED patients with sepsis from 4 hospitals in Utah^3,22,26,27^ to qualitative data addressing sepsis evaluation and treatment practices from semistructured physician interviews. We adhered to the Strengthening the Reporting of Observational Studies in Epidemiology (STROBE) and Consolidated Criteria for Reporting Qualitative Research (COREQ) reporting guidelines. The study was approved by the Intermountain Health Institutional Review Board. Quantitative analyses were conducted under waivers of informed consent. Interview participants provided written informed consent. eAppendix 1 in Supplement 1 provides additional details regarding study setting, participant eligibility criteria, study team positionality, data collection, and qualitative and quantitative analysis methods.

### Patient and Physician Participants

Adult patients without trauma (aged ≥18 years) presenting to a study hospital^28^ from July 1, 2013, to January 31, 2017, were eligible for quantitative analyses during the first ED visit in which they met Sepsis-3 consensus criteria^8^—including receipt of intravenous antimicrobials or enteral oseltamivir phosphate, fidaxomicin, or vancomycin hydrochloride for suspected or confirmed infection—prior to ED departure. Patient follow-up ended 30 days after ED arrival. ED attending physicians—followed up for as long as 43 months for quantitative analyses—were included in the analytic cohort if they treated 20 or more eligible patients with sepsis during the 2013-2017 study period. Physicians were eligible to participate in qualitative interviews conducted between May 17, 2022, and July 28, 2023, if they remained employed at a study hospital and their estimated mean door-to-antimicrobial time was in the fastest or slowest quartile compared with other physicians, with planned enrollment of as many as 10 physicians per quartile or until thematic saturation was achieved.^29^

### Quantitative Data Collection

As previously described,^3,22,26,27^ data extracted from the Intermountain Health electronic data warehouse was supplemented by structured manual abstraction for reconciliation of missing, outlying, or implausible data. The patient’s primary ED attending physician was identified for 98.4% of records using an algorithm^27^ integrating documentation authorship, billing, and ED patient tracking data (the eMethods in Supplement 1 provides detailed methods and validation). Manual medical record review identified the ED physician for the remaining cases. Using all available data, trained abstractors also applied standardized criteria and validated methods to adjudicate whether infection was actually present in the ED.^26^ eAppendix 1 and eFigure 1 in Supplement 1 provide additional details regarding infection presence adjudication and other aspects of quantitative data collection.

Self-reported demographic data collected from electronic health records included age, sex, and race and ethnicity. Race and ethnicity are reported as Hispanic or Latino ethnicity or race other than White (including American Indian or Alaska Native, Asian, Black or African American, Native Hawaiian or Other Pacific Islander, or multiracial) or as non-Hispanic or non-Latino White. These data were placed in binary categories to allow characterization of the proportion of racial and ethnic minoritized individuals in the patient cohort.

Patients were considered overtreated if they met sepsis criteria prior to ED departure—including receiving antimicrobials in the ED for suspected or diagnosed infection—but had infection ruled out on final retrospective adjudication using all available data (eMethods in Supplement 1). Patient-level spectrum scores were calculated as the sum of the spectrum score (eAppendix 3 in Supplement 1) for each unique antibiotic administered in the ED.^30^

### Qualitative Data Collection

A draft interview script created by topical and methods experts (I.D.P., J.B., and S.M.B.) was pilot tested and iteratively revised. The final semistructured interview script (eAppendix 2 in Supplement 1) elicited general knowledge and opinions about sepsis diagnosis and management and applied the critical decision variant of cognitive task analysis^31^ referenced to a self-selected “difficult” case of possible sepsis to elicit physicians’ approach to sepsis evaluation and treatment. Questions and probes paid particular attention to the management of uncertainty and task prioritization.

To avoid bias, the interviewer (J.B.) was masked to participants’ door-to-antimicrobial time. Interviews occurred via videoconference with a target duration of 30 to 40 minutes. Audio-recorded interviews were professionally transcribed.

### Statistical Analysis

#### Quantitative Data Analysis

Details of statistical methods are provided below and in eAppendix 1 in Supplement 1. In summary, we characterized physician door-to-antimicrobial time variation using a linear mixed-effects model. Separately, the primary and secondary analyses examined whether physician-level variation in door-to-antimicrobial time was associated with overtreatment using a joint modeling approach that simultaneously modeled door-to-antimicrobial time and overtreatment, linking the 2 submodels through a shared physician-level random effect.

Characterization of physician-level door-to-antimicrobial time variation used a linear mixed-effects model for door-to-antimicrobial time incorporating an identity link, patient-level fixed-effects (study hospital and patient age, sex, race and ethnicity other than non-Hispanic or non-Latino White, ED arrival via ambulance, weighted comorbidity score,^32,33^ infection source, preferred language, the Mortality in Emergency Department Sepsis score,^34,35^ nighttime ED arrival, shock on ED arrival, pooled triage acuity score,^36^ and year of ED arrival), and a random intercept for physician. A likelihood ratio test evaluated the hypothesis that door-to-antimicrobial time varied across physicians by comparing models with and without the physician-level random effect.^37^ The intraclass correlation for the random physician effect estimated the physician-attributable proportion of door-to-antimicrobial time variation. Empirical best linear unbiased predictions of the physician random effects (termed *physician-estimated mean door-to-antimicrobial times*) were measured and physician-level variation quantified via the 95% estimation interval for the average physician-estimated mean door-to-antimicrobial time and via graphical presentation of physician-estimated means for a typical patient (ie, mean values for continuous covariates and the most common level for categorical covariates). We evaluated the association of physician characteristics (experience, sex, and residency training) with practice by adding physician-level fixed effects for these parameters to the mixed-effects model.

The primary analysis used a joint mixed-effects shared parameter model to estimate the association between the primary exposure, physician mean door-to-antimicrobial time structured as a latent physician-level parameter, and the primary outcome, physician-level overtreatment probability. In this model, conditional on patient-level fixed-effects covariates as above, the probability of overtreatment was allowed to depend on physicians’ latent mean door-to-antimicrobial times, modeled as a random effect. The joint model included a Bernoulli submodel for the probability of overtreatment and a gaussian submodel for door-to-antimicrobial time. Analysis of the secondary outcome used an analogous approach substituting a gaussian submodel with an identity link for antibiotic spectrum. In both analyses, the joint model included a coefficient for the random intercept for the latent physician mean door-to-antimicrobial times that quantified the association of interest.^38,39,40,41^ Model estimates are reported as an adjusted odds ratio for the probability of overtreatment and as an adjusted mean change for antibiotic spectrum, along with model-derived 95% CIs and *P* values.

Quantitative data analysis occurred between 2021 and 2025. Statistical significance was defined by a 2-tailed *P* ≤ .05.

#### Qualitative Data Analysis

Analysis of transcribed interviews occurred from 2022 to 2025 and used a combination of inductive and deductive methods via a process consistent with development of a thematic analysis.^42,43^ Interviews were independently coded by 2 qualitative researchers (F.M.S. and M.B.M.) masked to participants’ door-to-antimicrobial time using an iteratively refined codebook. Analysis focused on 3 preselected code groups: clinical decision-making, care coordination, and sepsis care protocols. Selection was informed by the threshold model of decision-making under uncertainty,^44^ fuzzy trace theory,^45,46^ and the dual-process model of clinical decision-making.^47^ Illustrative quotations informative of antimicrobial initiation behavior were selected before the physician door-to-antimicrobial time quartile was unmasked, after which comparative interpretation for each code was iteratively synthesized by the analysis team.

## Results

Among 9180 patients who met sepsis criteria in the study EDs between 2013 and 2017 and were included in quantitative analyses (eFigure 2 in Supplement 1), the median age was 63 (IQR, 48-75) years, 4635 (50.5%) were female and 4545 (49.5%) were male, and 3540 (38.6%) received antimicrobials in the ED more than 3 hours after ED arrival. In terms of race and ethnicity, 1510 patients (16.4%) were of Hispanic or Latino ethnicity or race other than White and 7670 (83.6%) were non-Hispanic or non-Latino White. On final retrospective adjudication, 778 patients (8.5%) were determined to not be infected at the time of ED arrival and thus not have sepsis (Table 1 and eTable 1 in Supplement 1). Analyses included 88 ED attending physicians who cared for at least 20 eligible patients with sepsis during the study period, with 17 physicians (19.3%) female and 71 (80.5%) male. Median age was 39 (IQR 35-49) years, and median post–medical school experience was 11 (IQR 7-21) years. (Table 2 and eTable 2 in Supplement 1). The median number of encounters with patients with sepsis per included physician was 105 (IQR, 75-129). The median physician-level door-to-antimicrobial time was 155 (IQR, 138-172) minutes and ranged from 89.5 to 218.0 minutes.

**Table 1.** Patient Demographic and Clinical Characteristics by Door-to-Antimicrobial Time

Characteristic	Overall (N = 9180)	Door-to-antimicrobial time		*P* value^a^
		≤3 h (n = 5640)	>3 h (n = 3540)	
Age, median (IQR), y	63 (48-75)	63 (50-76)	62 (47-75)	.003
Sex, No. (%)				
Female	4635 (50.5)	2710 (48.0)	1925 (54.4)	<.001
Male	4545 (49.5)	2930 (52.0)	1615 (45.6)	
Race and ethnicity, No. (%)				
Hispanic or Latino ethnicity or race other than White^b^	1510 (16.4)	918 (16.3)	592 (16.7)	.06
Non-Hispanic or non-Latino White	7670 (83.6)	4722 (83.7)	2948 (83.3)	
Weighted Elixhauser comorbidity score, median (IQR)	0 (0-6)	0 (0-12)	0 (0-0)	<.001
English as preferred language	8679 (94.5)	5357 (95.0)	3322 (93.8)	.02
Brought to ED by ambulance, No. (%)	2746 (29.9)	1938 (34.4)	808 (22.8)	<.001
Nighttime ED arrival (12 am to 6:59 am), No. (%)	1032 (11.2)	723 (12.8)	309 (8.7)	<.001
Triage acuity score, No. (%)				
Resuscitation	138 (1.5)	126 (2.2)	12 (0.3)	<.001
Emergent	5077 (55.3)	3383 (60.0)	1694 (47.9)	
Urgent	3817 (41.6)	2039 (36.2)	1778 (50.2)	
Semiurgent or nonurgent	148 (1.6)	92 (1.6)	56 (1.6)	
MEDS score, median (IQR)^c^	6 (5-9)	8 (5-9)	6 (3-8)	<.001
SOFA score, median (IQR)^d^	4 (3-6)	4 (3-6)	3 (3-5)	<.001
Shock present on ED arrival, No. (%)	825 (9.0)	586 (10.4)	239 (6.8)	<.001
Lactate level >18 mg/dL, No. (%)	3569 (38.9)	2417 (42.9)	1152 (32.5)	<.001
ED-diagnosed source of infection, No. (%)				
Pulmonary	3628 (39.5)	2461 (43.6)	1167 (33.0)	<.001
Urinary	1717 (18.7)	1018 (18.0)	699 (19.7)	
Other	3835 (41.8)	2161 (38.3)	1674 (47.3)	
Infection absent on final adjudication, No. (%)	778 (8.5)	391 (6.9)	387 (10.9)	<.001
Admission to ICU from ED, No. (%)	2856 (31.1)	2069 (36.7)	777 (21.9)	<.001
30-d mortality, No. (%)	796 (8.7)	538 (9.5)	258 (7.3)	<.001

Abbreviations: ED, emergency department; ICU, intensive care unit; MEDS, Mortality in Emergency Department Sepsis; SOFA, Sequential Organ Failure Assessment.

SI conversion factor: To convert lactate to mmol/L, multiply by 0.111.

^a^ *P* values are reported at the patient level and do not account for clustering by physician.

^b^ Race other than White includes American Indian or Alaska Native, Asian, Black or African American, Native Hawaiian or Pacific Islander, and multiracial or other.

^c^ Scores range from 0 to 27, with higher scores indicating a higher risk for mortality.

^d^ Scores range from 0 to 24, with higher scores indicating more severe organ failure.

**Table 2.** Physician Demographic Characteristics by Physicians’ Estimated Mean Door-to-Antimicrobial Time Quartile

Characteristic	Overall (N = 88)	Mean door-to-antimicrobial quartile			
		First (shortest) (n = 22)	Second (n = 22)	Third (n = 22)	Fourth (longest) (n = 22)
Eligible patient encounters, median (IQR)	105 (75-129)	112 (49-147)	98 (50-126)	113 (78-124)	103.5 (79-115)
Age, median (IQR), y^a^	39 (35-49)	41 (37-48)	39 (34-48)	41 (35-48)	39 (35-55)
Sex, No. (%)					
Female	17 (19.3)	4 (18.2)	10 (45.5)	1 (4.5)	2 (9.1)
Male	71 (80.7)	18 (81.8)	12 (54.5)	21 (95.5)	20 (90.9)
Time since medical school, median (IQR), y^a^	11 (7-21)	12 (8-20)	11 (7-21)	11.5 (7-20)	9.5 (7-25)
Completed EM residency, No. (%)	74 (84.1)	19 (86.4)	19 (86.4)	19 (86.4)	17 (77.3)

Abbreviation: EM, emergency medicine.

^a^ Measured at the time the physician cared for their first eligible patient during the study period.

After accounting for patient characteristics, the door-to-antimicrobial times for patients treated from 2013 to 2017 varied significantly across ED physicians (likelihood ratio test comparing models with vs without a physician-level random effect, *P* < .001). For a typical patient, the average physician-estimated mean door-to-antimicrobial time of 184 minutes had a 95% estimation interval of 146 to 222 minutes (Figure). The proportion of variation attributable to the physician (intraclass correlation) was modest at 5.1%. Physician sex, residency training, and years of experience were not associated with door-to-antimicrobial time (eTable 3 in Supplement 1).

**Figure.**
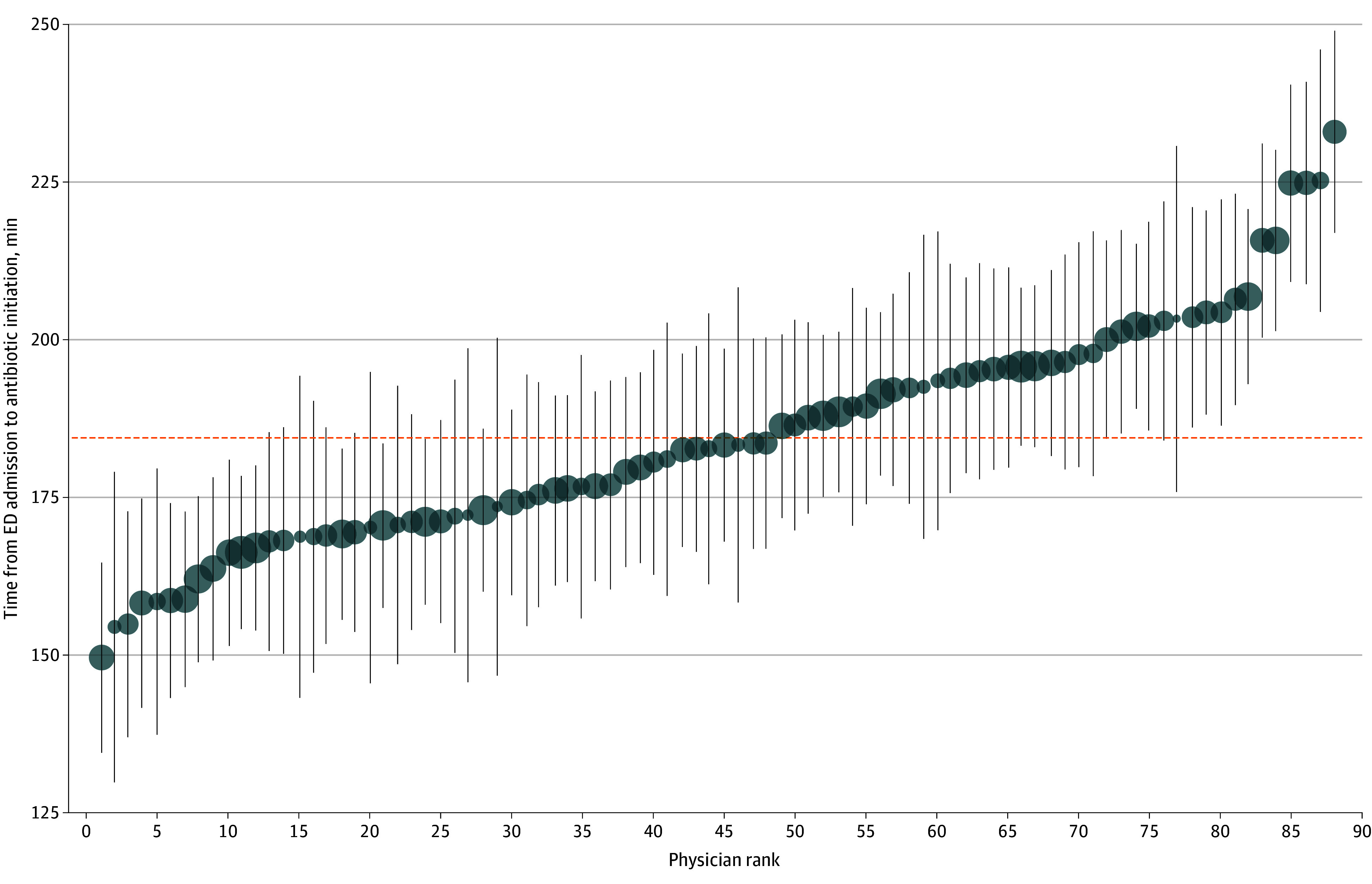
Best Linear Unbiased Predictions (Physician-Estimated Mean) for Door-to-Antimicrobial Time Best linear unbiased predictions for typical patients with sepsis treated by 88 attending emergency department (ED) physicians in the ED are depicted (circles) with 95% prediction intervals (vertical lines). Physician rank is determined by door-to-antimicrobial time. Circle sizes are proportional to the number of eligible patients with sepsis cared for by each physician. The horizontal dotted line indicates the average physician-estimated mean door-to-antimicrobial time for all physicians.

In the primary analysis, the odds of overtreatment (patient received antimicrobials in the ED but was not infected on final adjudication) was not associated with physician door-to-antimicrobial time (adjusted odds ratio for every 10-minute increase in ED physician door-to-antimicrobial time, 0.98 [95% CI, 0.94-1.02]; *P* = .37). In the secondary outcome analysis restricted to patients receiving antibiotics (n = 9072), the spectrum score for antibiotics received in the ED was also not associated with ED physician door-to-antimicrobial time (adjusted increase in spectrum score for each 10-minute increase in ED physician door-to-antibiotic time, 0.049 [95% CI, −0.002 to 0.100] points; *P* = .06).

Eighteen physicians participated in research interviews conducted from 2022 to 2023, equally split between the fastest and slowest quartiles for estimated mean door-to-antimicrobial time (eTable 2 in Supplement 1). Table 3 provides illustrative quotations, with unedited quotations provided in eTable 4 in Supplement 1.

**Table 3.** Illustrative Physician Quotations by Topic Code and Door-to-Antimicrobial Quartile

Topic code	Quotation (study participant identification No.)	
	Faster door-to-antimicrobial quartile	Slower door-to-antimicrobial quartile
Clinical decision-making	Some of that will be your clinical gestalt … you can recognize kind of the sick and not sick. (101)	I’m always not ruling out sepsis as a possibility … I’m always keeping sepsis on the differential…. I will continue to work down the sepsis algorithm, until there’s something else that may come up as a more probable explanation. (120)
	I think when you walk in the room, a lot of times you can tell just by looking at a patient. (104)	I can’t tell you I have a great algorithm, I have enough experience that I trust my gut to say, OK. That person is sick. Let’s give him antibiotics now…. I wish I could put a nice checklist for you on it with a nice outline, but it’s a little less precise than that. (116)
	I think experienced clinicians do develop an ability to walk into a room and just have a gestalt that this patient is sick.… Your decision has been made and you put the train on the track, so to speak, and you’re not going to take it off those tracks until the patient convinces you kind of emphatically that they’re not sick. (113)	Again, walking in the room and talking to the patient is my best sense. It’s hard to describe. But after 10 years of clinical practice, I feel like I have a sense within 30 seconds of how sick someone is. (107)
	I would say it’s, from the outset, it’s really … a gestalt of their clinical appearance, what the person looks like in bed. Are they looking sick? A person who looks sick often is going to be sick. (118)	
Multidisciplinary care coordination	Keeping my concern about that potential sepsis patient in my mind, and keeping it in the nurses’ mind to say, “Hey. Even though things look good, please let me know if anything changes” … so really when it’s busy, it’s rallying the team to, again, using our nurses to recognize sepsis in a similar way that I recognize sepsis. (101)	Delays come all over the place. Once I put antibiotics in, sometimes the nurse is busy somewhere else. Sometimes we have to get it from central. It’s not actually in our Pyxis and that can take some time…. You order shotgun everything at once and then you are at the mercy of the Phleb on whether the labs come back soon. The nurse on how quickly she can get the viral swab off. The x-ray tech on how soon they can get to x-rays…. If someone is really sick then I’ll go find the nurse myself and say hey, this patient needs our attention. (103)
	We all kind of huddle outside the room and talk about the plan at one time. (106)	And so you’re like, hey, I put this order in half an hour ago, “how come you haven’t been given?” And the nurse is like … “pharmacy hasn’t brought it up yet.” (114)
	We’ve sort of tried to train the nurses that if you see somebody who looks sick or unstable, just let us know because then we can come to the room and layout kind of what our plan is going to be, a plan of attack in order of operations so that then everybody is on board with what needs to happen at each step of the way. (109)	If I’ve decided to do antibiotics, I always communicate directly with the nurse, like I said, are experienced self-motivated nurses, I probably won’t go beyond that. Because I know that they will themselves talk to the pharmacist and call the inpatient pharmacy and get things going. I’ll ask the nurse to kind of go in there early, try to get things done early. And so I’ll usually involve the pharmacists for them to say, I need antibiotics, can you make sure they’re coming from pharmacy and that you deliver into the room and kind of prompt the nurse to get them started and here’s why. (120)
	On a patient like this, I probably have the nurse in the room anyway because we are doing a lot of things and trying to figure things out. I told the nurse, here’s what I’m going to order. Let’s administer this antibiotic after you get the blood cultures. (111)	
	I might talk to the nurse and just say hey, I ordered antibiotics for this patient. Will you look for them when they come from the pharmacy? They need to be infused…. Usually if they understand the reasoning behind, then they understand that you want them sooner rather than later. (118)	
Protocols and care bundles	Well, particularly with the EMR, there’s so many places that I would have to go, to click boxes to get things done. And the sepsis bundle allows me to pull it all on the one page and click what I don’t want. (105)	The protocol meant that we didn’t really miss people with sepsis. Here, we do have protocols and bundles and they’re helpful. (103)
	I find them helpful and distracting, at the same time…. I think bundles are more helpful in making sure that we don’t omit important care … they treat everyone one size fits all…. We’ll do some patients harm, and so people get frustrated by that. (106)	I like them. I’ve seen they improve outcomes across different care spaces that might struggle…. Bundles and protocols help empower each member of the team to impact outcomes. (107)
	I’m middle of the road. I don’t have strong feelings about it…. I have some concerns when it pulls … resources towards a patient that doesn’t really need them and away from others. (108)	I like protocols that kind of decreases variability. I do not like bundles because the bundle is like a one size shoe. For a lot of different sized feet. (110)
	I think it’s really helpful for antibiotic choices, especially when we’re at the smaller facilities where we don’t have an ED pharmacist on there because then we don’t have to think about what do I need to give this person? … it takes one more cognitive exercise off of your list to do that. (109)	Generally I’m in favor of them. I think they’re good to help remind us, hey, we need to think about this, we need to think about this early … there are definitely nuances that bundles don’t necessarily take into account or appreciate. (114)
	Love them. I think it’s great. One thing I really like about it is it’s hard for me personally to stay on the most current literature of everything that I’m treating…. I have faith that, whoever is putting these in here is keeping them up to date on what they think is proper and appropriate. (111)	I think it simplifies things a little bit when it comes to some of the treatment decisions. I’d say I’m for them. I’m for protocols in general. (115)
		So, I am Jekyll and Hyde on this subject…. I really like the sepsis workup bundle…. I find that the treatment recommendation just to be a little more frustrating…. It’s just because there’s no certainty that they’re there…. I don’t like doing something just for the sake of doing something, but I appreciate that we probably should be. (116)

Abbreviations: ED, emergency department; EMR, electronic medical record.

Physicians reported substantial reliance on clinical gestalt for sepsis identification and clinical decision-making that was generally similar across door-to-antimicrobial quartiles (Table 3). However, some physicians in the slower door-to-antimicrobial quartile seemed slightly more likely to supplement heuristics with deliberate algorithmic thinking.

Physicians in both quartiles emphasized the multidisciplinary, team-based nature of ED sepsis care delivery (Table 3). Teamwork descriptions by physicians who were slower to administer antimicrobials described engaging in ad hoc communications with other ED team members later in the encounter after confirming suspicion of sepsis and ordering antimicrobials. These physicians viewed patient care as vulnerable to systemic factors and inefficiencies, which they lacked agency to prevent and instead addressed reactively and stepwise. By contrast, physicians who administered antimicrobials faster described concurrent clinical task execution and used proactive strategies and communication to coordinate with the ED care team, including sharing responsibility with ED nurses when trying to identify sepsis early in the ED encounter (Table 3).

Most physicians believed care protocols reduced cognitive loads, ensured access to current evidence-based information, and helped avoid omission of important steps in sepsis diagnosis and treatment (Table 3). Physicians in the slower antimicrobial quartile expressed slightly more ambivalence about bundles, however, describing a negative perception of sepsis protocols as “one size fits all” and noting concerns regarding interruptions to cognitive flow (“distracting”).

## Discussion

In this large, multicenter cohort study using quantitative and qualitative analysis, door-to-antimicrobial time among ED physicians varied significantly. Physicians with faster door-to-antimicrobial times, however, did not exhibit significantly increased rates of overtreatment (antimicrobials given in the ED but infection not present in the ED on retrospective adjudication) or administer broader-spectrum antibiotics. In interviews conducted from 2022 to 2023, physicians who had been in the fastest quartile for door-to-antimicrobial time in 2013 to 2017 described using parallel task execution and proactive coordination of the multidisciplinary care team during evaluation and treatment of patients with potential sepsis, while physicians in the slower quartile reported a more reactive and stepwise approach. Physicians in the fastest door-to-antimicrobial quartile also placed slightly more emphasis on heuristics and pattern recognition (ie, type 1 clinical decision-making) over deliberate algorithmic assessment (ie, type 2 clinical decision-making)^47^ regarding the presence and management of sepsis. Sepsis care bundles occasioned more concern regarding the standardization and potential harms among physicians with slower door-to-antimicrobial times.

Considering the central role of timely administration of antimicrobials in optimizing sepsis outcomes,^1,2,3,4,5,6,7^ identification of nonpatient factors that aid or impede this therapy’s delivery is critical to systematic sepsis care improvement. This study confirms previous pilot data^25^ indicating that physician door-to-antimicrobial timing varies. Physicians’ demographic and training characteristics were not associated with their door-to-antimicrobial times. However, qualitative investigation illuminated differences in how physicians approached sepsis evaluation and treatment. Physicians consistently highlighted the complex, team-based nature of sepsis care, but physicians with slower door-to-antimicrobial times tended to view themselves as cogs within a care delivery system, the operation of which they had limited power to modify. While these physicians incorporated more algorithmic decision-making alongside heuristics in their sepsis care, this did not appear to be a conscious choice made to improve diagnostic certainty and reduce overtreatment. Physicians in the fastest quartile for door-to-antimicrobial time, by comparison, appeared to feel empowered to catalyze sepsis care via active coordination of the multidisciplinary team. These physicians reported use of proactive, efficient team-based communication to overcome barriers that can contribute to delays in the identification and treatment of sepsis. They also organized care of sepsis as a set of parallel processes, in contrast to physicians in the slowest door-to-antimicrobial time quartile, who more often saw sepsis evaluation and treatment as a sequential, progressive process.

These insights suggest outlooks and strategies that individual physicians could adopt to aid sepsis care as well as potential system-based interventions to accelerate and synchronize multidisciplinary teams’ sepsis evaluation and treatment.^14,48,49^ While care team huddles can reduce time to antimicrobial administration,^14^ our data suggest clinician-driven asynchronous communication and care coordination contribute to prompt antimicrobial administration. The fact that ED physicians with slower door-to-antimicrobial times seemed resigned to accept care delivery obstacles suggest that interventions promoting clinicians’ confidence in their ability to effectively direct time-critical care by multidisciplinary teams (ie, self-efficacy^50^) may be helpful for some physicians.

Our findings also provide objective data to address concerns regarding the potential for harm from quality improvement efforts in sepsis care—namely, that efforts to accelerate antimicrobial treatment may drive administration of unnecessary antimicrobials or unnecessarily broad antimicrobials.^51,52,53^ Such a spillover effect could lead to clinically important patient-level harms, since complications—including allergic reactions, kidney injury, and *Clostridioides difficile* colitis—are frequent with antimicrobial therapy.^15,54^ Adverse health system-level consequences, including increased antimicrobial resistance and cost, are also of concern.^55^ Fortunately, we found that physicians with practice patterns involving earlier antimicrobial administration did not have more patients for whom infection was ruled out on final adjudication and did not use broader-spectrum antibiotics. Since patients’ assignment to an ED physician is substantially random, ED physicians are an admittedly imperfect instrument for care delivery patterns.^56^ This property, combined with robust statistical methods to account for persistent confounding, strengthens causal inference from our analysis. Our data also align with a conceptually similar study published in 2022 that found that hospital-level practice shifting to earlier antimicrobial administration was not associated with increased antimicrobial treatment, days of therapy, or spectrum.^18^

### Strengths and Limitations

Strengths of our study include the analysis of patients and physicians from multiple hospitals with variable sizes and resources, a well-characterized cohort of ED patients, and linkage of robust causal inference methods with qualitative analysis. However, our study has several limitations. Initiation of antibiotic therapy for sepsis has accelerated during and since our quantitative practice data were collected between 2013 and 2017,^57^ and temporal trends toward faster initiation of antimicrobial treatment overall may be one reason this analysis found a lower proportion of physician-attributable variation in antimicrobial timing compared with the pilot study.^25^ Physicians’ sepsis care practices and beliefs may have evolved during the several-year time gap between practice measurement and interviews. Physicians who participated in interviews may have differed from those who declined participation or were unavailable due to leaving the study health system, retirement, or other factors. The SEP-1 sepsis care quality metrics^58,59^ and the Sepsis-3 criteria used to identify patients eligible for this analysis were released in 2015 and 2016, respectively, and differ from the older sepsis metrics and definitions that informed bedside clinical care during a portion of the period for which quantitative data were collected. Statistical models adjusted for a comprehensive set of patient characteristics, but a nonlinear association of physician antimicrobial time with overtreatment probability, study site differences, or residual confounding may have affected our findings. We focused on patient-level process-of-care spillover effects^55^ and were unable to investigate whether care patterns leading to earlier initiation of antimicrobial treatment were associated with care or outcomes among patients without sepsis (eg, diagnostic delays), patient-centered outcomes among patients with sepsis (eg, increased *C difficile* infections), or the incidence of drug-resistant infections. We also could not assess whether earlier antimicrobial initiation practices were associated with a shift toward more possible (rather than probable or definite) infections among patients not classified as overtreated (ie, infection not ruled out). Use of cognitive task analysis anchored to real patient cases reduced but did not eliminate the possibility of recall bias during physician interviews. The included ED physicians were predominantly male, although the study cohort’s demographic characteristics are broadly proportional to the US emergency medicine physician workforce, which is 28% female.^60^ Finally, it is possible that our results may not be generalizable to other health systems or geographic regions.

## Conclusions

In this explanatory mixed-methods study, we confirmed significant physician-level variation in door-to-antimicrobial time when treating patients with sepsis. Physician practice patterns characterized by earlier antimicrobial administration were not associated with increased antimicrobial overtreatment. Physicians who had the fastest door-to-antimicrobial administration times actively engaged in proactive, timely communication and coordination with the multidisciplinary care team to organize parallel task execution and felt empowered to overcome system-level obstacles to prompt sepsis evaluation.

## Notes

Supplement 1. **eAppendix 1.** Supplementary Methods **eAppendix 2.** Physician Interview Script **eAppendix 3.** Antibiotic Spectrum Scores **eTable 1.** Patient Characteristics by Physician-Estimated Door-to-Antimicrobial Time Quartile **eTable 2.** Physician Demographics by Interview Eligibility and Participation **eTable 3.** Association of Physician Characteristics With Antimicrobial Timing **eTable 4.** Unselected Interview Quotations Related to Preselected Codes **eFigure 1.** Criteria for Retrospective Adjudication of ED Infection Presence **eFigure 2.** Patient and Physician Participant Inclusion-Exclusion Diagram
eReferences

Supplement 2.
Data Sharing Statement
