# Supplementary material for: Physician Variation in Early Sepsis Management
Source: JAMA Netw Open. 2026 Feb 13;9(2):e2556945. doi: 10.1001/jamanetworkopen.2025.56945 (PMC12905659; doi:10.1001/jamanetworkopen.2025.56945)
Supplement: Supplement 1. — eAppendix 1. Supplementary Methods eAppendix 2. Physician Interview Script eAppendix 3. Antibiotic Spectrum Scores eTable 1. Patient Characteristics by Physician-Estimated Door-to-Antimicrobial Time Quartile eTable 2. Physician Demographics by Interview Eligibility and Participation eTable 3. Association of Physician Characteristics With Antimicrobial Timing eTable 4. Unselected Interview Quotations Related to Preselected Codes eFigure 1. Criteria for Retrospective Adjudication of ED Infection Presence eFigure 2. Patient and Physician Participant Inclusion-Exclusion Diagram eReferences [file jamanetwopen-e2556945-s001.pdf]

Supplementary Online Content

Peltan ID, Groat D, Butler J, et al. Factors in physician variation in early sepsis management. *JAMA Netw Open.* 2026;9(2):e2556945. doi:10.1001/jamanetworkopen.2025.56945

eAppendix 1. Supplementary Methods
eAppendix 2. Physician Interview Script
eAppendix 3. Antibiotic Spectrum Scores
eTable 1. Patient Characteristics by Physician-Estimated Door-to-Antimicrobial Time Quartile
eTable 2. Physician Demographics by Interview Eligibility and Participation
eTable 3. Association of Physician Characteristics With Antimicrobial Timing
eTable 4. Unselected Interview Quotations Related to Preselected Codes
eFigure 1. Criteria for Retrospective Adjudication of ED Infection Presence
eFigure 2. Patient and Physician Participant Inclusion-Exclusion Diagram
eReferences

This supplementary material has been provided by the authors to give readers additional information about their work.

© 2026 Peltan ID et al. *JAMA Network Open*.

eAppendix 1. Supplementary Methods

Setting
The study enrolled patients presenting to the ED of one of four Intermountain Health hospitals in Utah. Study hospitals included two community hospitals, regional referral hospital, and a tertiary teaching and trauma center. Annual ED visits were approximately 22,000-28,000 at the community and regional referral hospital and 87,000 at the tertiary hospital. Study hospitals used a shared sepsis protocol[1] and, during the quantitative data capture period, employed ongoing sepsis quality improvement efforts but had no substantive shifts in overall practices.

Patient subject eligibility criteria
Adult patients (age ≥18 years) presenting to a study hospital from July 1, 2013 to January 31, 2017 were eligible for study inclusion if they met international Sepsis-3 consensus criteria for sepsis[2] prior to ED departure. Specifically, patients were considered to meet sepsis criteria if they exhibited acute organ failure (defined by a Sequential Organ Failure Assessment score[3] ≥2 points above baseline) plus suspected or confirmed infection (defined by collection of blood cultures plus administration of intravenous antimicrobials or enteral oseltamivir, fidaxomicin, or vancomycin). Patients who had a trauma team activation or infection ruled out in the ED we excluded, as were subsequent ED visits by already-included patients and patients whose primary ED attending physician treated fewer than 19 other eligible sepsis patients.

Physician interview subject recruitment
After pre-notification via ED clinical leadership, ED physicians eligible for participation in study interviews were identified based on their predicted mean door-to-antimicrobial time (top or bottom quartile compared to other physicians included in the analysis). Eligible physicians were invited in-person or via email to participate in study interviews. To prevent recruitment and response bias, potential subjects' relative antimicrobial initiation behavior was not disclosed to the subjects or study team during recruitment or interviews. Interviews were conducted via videoconference. In addition to the physician and interviewer, a research coordinator facilitated interview recording and use of the videoconferencing software during some interviews. Interviews were conducted between May 17, 2022 and June 28, 2023.

Positionality statement
The research team is based in the United Statues and consisted of clinical and non-clinical research personnel with expertise in emergency medicine, infectious disease, critical care, psychology, sepsis care, biostatistics, implementation science, qualitative research methods, and decision science. The female doctoral-level psychologist (JB) who conducted interviews had no prior relationship with study subjects. The principal investigator (IDP) is an intensive care physician at Intermountain Medical Center (where some physicians who participated in research interviews also practice) and leads a research program that investigates the optimization of sepsis care, including the positive and negative consequences of early antimicrobial treatment and how health system and clinician factors influence sepsis care delivery. In general, the research team considers that prompt administration of appropriate antimicrobials is a key component of optimal care for patients with sepsis.

Quantitative data collection
Data extracted from the Intermountain Healthcare electronic data warehouse was supplemented by manual abstraction for missing data, outlying or implausible values as previously described.[4-7] Trained abstractors also applied standardized criteria to identify the source of infection diagnosed by the ED clinician and make a final retrospective adjudication regarding whether infection was present in the ED and if so, the infection's source.[6] Detailed methods for this adjudication have been previously reported.[6] In brief, adjudication of the final presence and source of infection employed all available documentation — including discharge summaries, physician notes, post-discharge outpatient care data, microbiology results, diagnostic imaging, relevant laboratory and molecular diagnostic testing — to classify the presence of infection. In general, infection was considered to be at least possibly present (i.e., not ruled out) if patients at minimum exhibited a clinical syndrome consistent with acute infection with sufficient clinical concern for the treating clinical team to administer a full course of antimicrobial therapy (eFigure 1).

We identified the ED attending physician who had primary responsibility for patients' ED care using a deterministic algorithm integrating clinical documentation authorship, billing records, and data from ED patient

© 2026 Peltan ID et al. *JAMA Network Open*.

tracking “dashboards.[7] The following seven rules were applied sequentially, and the algorithm halted as soon as a rule designated the primary ED attending physician:

1. Designate the ED attending physician as the certifying author of the first ED physician note if this person matches both the first ED attending physician identified on the ED tracking dashboard and the billing clinician.
2. Designate the ED attending physician as the first ED attending physician identified in ED tracking dashboard data if this person matches the billing clinician and is also the certifying author of the first or second ED physician note.
3. Designate the ED attending physician as the certifying author of the first ED physician note if there was only one ED physician note and valid data for the first ED attending physician was missing from both the ED tracking dashboard data and the billing ED clinician data (e.g., data are missing or identify an inpatient physician rather than ED clinician).
4. Designate the ED attending physician as the first attending physician identified in ED tracking board data if this person matches the billing clinician, there is only one ED physician note, but the certifying author of this note is missing or invalid (e.g., data are missing or identify an inpatient physician rather than ED clinician).
5. Designate the ED attending physician as the certifying author of the first ED physician note if this person matches the ED tracking board data for first ED attending physician and there was only one ED physician note.
6. Designate the ED attending physician as the certifying author of the first ED physician note if this person matches the ED billing clinician and there was only one ED physician note.
7. Do not designate an ED attending physician designated via the algorithm. Identify ED attending physician via manual review of the medical record.

The algorithm yielded an automated primary ED attending physician designation for 9036 (98.4%) of analyzed ED encounters and required manual adjudication for the remaining 144 (1.6%). In a subset (N=817) of analyzed encounters for which the algorithm identified an ED attending physician and data were available from an independent sepsis registry, the algorithm-generated physician assignment for 815 (99.8%) encounters agreed with the physician manually assigned for the registry by trained nurse abstractors.

Covariate data sources and definitions

Vital signs and the triage acuity score[8] were obtained from the first-recorded ED values. Race and ethnicity are reported to allow characterization of the minoritized proportion of the patient cohort and are classified from patient self-report recorded in the electronic medical record as “Hispanic/Latino or race other than White” or “White, non-Hispanic/Latino.” Race other than White includes American Indian or Alaska Native, Asian, Black or African American, Native Hawaiian or Pacific Islander, and multiple or other race. Shock on ED arrival was defined as systolic blood pressure <90 mmHg or mean arterial pressure < 65 mmHg. Patients’ comorbidity burden was based on a weighted score based on Elixhauser comorbidities.[9,10] We dichotomized preferred language as English versus other. Nighttime ED arrival was defined as midnight to 6:59 AM. Source of infection was categorized as pneumonia, urinary tract infection, or other. The Mortality in ED Sepsis (MEDS) score was calculated as previously described.[11,12]

Qualitative data collection and interview script creation

After initial development by investigators with expertise in sepsis care, emergency and critical care medicine, and qualitative research, a draft script for semi-structured interviews was piloted with three ED physicians, ineligible to participate in the interviews, and iteratively revised with their input for clarity, topical focus, comprehensiveness, and brevity. The final interview script (eAppendix 2) solicited general knowledge and opinions about sepsis diagnosis, management, and metrics and applied the critical decision variant of cognitive task analysis[13] referenced to a self-selected “difficult” case of possible sepsis to elicit physicians’ approach to sepsis evaluation and treatment with particular attention to the management of uncertainty and task prioritization.

Qualitative interviews occurred via videoconference. To avoid biasing subject responses, the interviewer — a female doctoral-level psychologist and expert qualitative researcher (JB) — was masked to subjects’ door-to-antimicrobial time. A professional service generated naturalized, deidentified transcriptions of the audio-recorded interviews.

© 2026 Peltan ID et al. *JAMA Network Open*.

Quantitative data analysis
Statistical significance was defined by a two-tailed p-value ≤0.05. Data analysis employed Stata version 16.1 (StataCorp, College Station, TX), R version 4.1.0 (R Foundation for Statistical Computing, Vienna, Austria), and SAS version 9.4 (SAS Institute, Cary, NC). No participants were lost to follow up. There were no missing values in the analysis dataset.

Descriptive analyses
Descriptive comparisons employed Wilcoxson rank-sum tests for dichotomous comparisons of continuous variables between two groups, and, as appropriate, either Fisher's exact test or chi-squared test for categorical variables.

Statistical modeling overview
Our primary research question was whether physician practice patterns involving faster antimicrobial administration for patients with sepsis were associated with increased antimicrobial overtreatment. The primary and secondary analyses investigated whether physician-level variation in door-to-antimicrobial time was associated with overtreatment using a joint modeling approach. This approach modeled physician-level door-to-antimicrobial time and physician-level overtreatment outcomes simultaneously, linking the two submodels via a shared physician-level random effect. While a simpler approach would have been to first summarize each physician's mean door-to-antimicrobial time and then relate these means to their overtreatment rates, the joint modeling approach allowed incorporation of the appropriate uncertainty in physicians' door-to-antimicrobial times (especially since the number of patients seen by each physician was variable) and allowed inclusion of patient-level covariates in analysis models.

We also wished to reconfirm prior pilot data[14] demonstrating variation among physicians in terms of their time to antimicrobial treatment for patients with sepsis. We used a linear mixed effects model to evaluate the existence of physician-level variation in door-to-antimicrobial time and quantified its extent. We also used this model to evaluate the relationship of physician characteristics to observed practice patterns.

Characterization of physician-level door-to-antimicrobial time variation
To investigate physician-level variation in door-to-antimicrobial time, we employed a linear mixed effects model incorporating door-to-antimicrobial time as the outcome, an identity link, a random intercept for physician, and fixed effects for a prespecified list of patient covariates (age, sex, race/ethnicity other than White/non-Hispanic, arrival to ED via ambulance, weighted comorbidity score, source of infection, preferred language, MEDS score, nighttime ED arrival, shock on ED arrival, pooled triage acuity score, and year of ED arrival). Study hospital was also included as a fixed effect since some ED physicians provided care at >1 study ED. A likelihood ratio test evaluated the hypothesis that door-to-antimicrobial time varies across physicians by comparing the linear mixed effect model with the physician-level random intercept to a simple linear regression model without a physician-level random effect. To quantify physician-level variation, the intraclass correlation (ICC) associated with the random physician effect estimated the proportion of total variation in door-to-antimicrobial time attributable to the physician. In addition, the empirical best linear unbiased prediction of the physician effects (termed "physician predicted mean door-to-antimicrobial time") were then estimated. We report the 95% prediction interval for the average physician-level predicted mean door-to-antimicrobial time — reflecting the expected range of true physician-specific predicted mean door-to-antimicrobial times across physicians — for a typical patient (i.e., a patient with average values for continuous covariates and the most common level for categorical covariates). We also graphically present the distribution for each physician's predicted mean door-to-antimicrobial time, including 95% confidence intervals associated with the physician-level means based on model-based standard errors of the random intercepts.

We assessed the association of physician experience (years since medical school), sex, and residency training (emergency medicine residency completed or not) with physician-level door-to-antimicrobial time by repeating this analysis after adding physician-level fixed effects for these parameters to the mixed effects model.

Joint mixed effects modeling (primary and secondary analyses)
The primary analysis employed a joint mixed effects shared parameter model with a Gaussian (normal) outcome submodel with an identity link for the door-to-antimicrobial time and a Bernoulli submodel with a logit link for the overtreatment outcome. The model included patient-level door-to-antimicrobial time, a patient-level dichotomous overtreatment indicator, a patient-level fixed effect for study hospital, prespecified patient-level covariates as above, and a physician-level random intercept. The physician-level random effect representing the ($b_{j[i]}$) latent parameter of physician mean door-to-antimicrobial time served as the "shared parameter" that induced correlation between the

© 2026 Peltan ID et al. *JAMA Network Open*.

two outcomes through their joint dependence on this latent effect. Evidence for an association between the physician-specific door-to-antimicrobial time and the probability of overtreatment was addressed via inference on the shared parameter through its coefficient λ in Eq 5.[15-18]

The joint model for the primary analysis had the form shown in Equations 1-5.

$$Y_{1i} = X_i\beta + \beta_0 + b_{j[i]} + \epsilon_i \quad \text{(Eq 1)}$$
$$\epsilon_i \sim N(0, \sigma_\epsilon^2), \quad \text{(Eq 2)}$$
$$b_{j[i]} \sim N(0, \sigma_b^2), \quad \text{(Eq 3)}$$
$$Y_{2i} \sim \text{Bernoulli}(p_i), \quad \text{(Eq 4)}$$
$$\text{logit}(p_i) = X_i\alpha + \lambda_0 + b_{j[i]}\lambda \quad \text{(Eq 5)}$$

Where $i = 1, \cdots, N$ designates the N patients and $j = 1, \cdots, J$ designate the J physicians, patients are nested within physicians, Y_{1i} denotes the door-to-antimicrobial time for patient i, X_i is the ith row of the $\boldsymbol{X}$ matrix for patient-level covariates, $j[i]$ indexes the ED physician who saw patient i, and Y_{2i} is an indicator variable for overtreatment of patient i. β_0 is the adjusted mean door-to-antimicrobial time across the physicians, ϵ_i is the residual door-to-antimicrobial time for patient i, p_i is the probability that patient i is overtreated. The $b_{j[i]}$ parameter, which defines the physician's adjusted mean door-to-antimicrobial time, links the Y_{1i} and Y_{2i} submodels, serving as a shared random effect. The coefficient λ of $b_{j[i]}$ in Eq 5 is the main target of inference and indicates the influence of the physician's adjusted mean door-to-antimicrobial time on the probability that the patients are over-treated.

Analysis of the secondary outcome used an analogous approach but applied a Gaussian model incorporating an identity link function for the antibiotic spectrum outcome. The joint model had the form shown above for Eq 1-3 but replaced Eq 5 with Eq 6-7 below, in which Y_{3i} denotes the total spectrum score for patient i and ε_i' is the residual total spectrum score for patient i. The coefficient θ of $b_{j[i]}$ in Eq 6 is the main target of inference and indicates the influence of the physician's adjusted mean door-to-antimicrobial time on the antibiotic spectrum score.

$$Y_{3i} = X_i\alpha + \theta_0 + b_{j[i]}\theta + \varepsilon_i' \quad \text{(Eq 6)}$$
$$\varepsilon_i' \sim N(0, \sigma_{\epsilon'}^2), \quad \text{(Eq 7)}$$

Implementation of joint model in SAS

We provide below representative SAS code used to fit the primary analysis model (PROC NLMIXED). For simplicity, both outcomes are assumed to be stacked in one long-format dataset with an indicator variable "dist" distinguishing continuous (Y_1) and binary (Y_2) for each patient. Separate submodels were fitted (linear and logistic mixed effects models) to provide starting values for $\beta_0, \beta, \alpha_0, \alpha, \lambda$, and the variance components.

```
/*--------------------------------------------------------------------------*/
/* Joint Gaussian-Logistic Mixed Model with Shared Random b_j[i] indicated by "b"*/
/*--------------------------------------------------------------------------*/

proc nlmixed data=jointdata qpoints=20 maxiter=100
                              maxfunc=2000 technique=newrap;
parms
beta0=144.64 beta1=0.1092 beta2=-9.8271 beta3=-2.4427
beta4=-29.8304 beta5=-27.1964 beta6=-31.6975 beta7=-15.6581
beta8=-0.6972 beta9=15.0456 beta10=2.987 beta11=-5.8207
beta12=-1.2971 beta13=-15.0556 beta14=-13.2133 beta15=48.2134
beta16=77.134 beta17=56.316 beta18=2.1761 beta19=-0.4308
beta20=-11.0664 beta21=-5.0197
sigmae=84.7249668 sigmab=19.43887857
alpha0=-1.9683
alpha1=0.005542 alpha2=0.04288 alpha3=0.06561 alpha4=0.1159
alpha5=-0.0974alpha6=0.03114 alpha7=0.3728 alpha8=0.004295
alpha9=0.5148 alpha10=-0.5212 alpha11=0.03455 alpha12=-0.0992
alpha13=0.05957 alpha14=0.1878 alpha15=-0.5801 alpha16=-0.7307
alpha17=-1.6081 alpha18=0.1644 alpha19=0.01248 alpha20=0.2398
alpha21=-0.1556 lambda=-0.01516
;
```

© 2026 Peltan ID et al. *JAMA Network Open*.

```
if dist = "Normal" then do;
    /* Corresponds to Eq 1: Continuous outcome submodel */
    mean = beta0 + beta1*age+beta2*sex_num+beta3*nonwhite_num+beta4*hosp_Alta+beta5*hosp_LDS+
beta6*hosp_Riverton+beta7*arrival_EMS+beta8*elix_score_vw+beta9*source_other+
beta10*source_Urinary+beta11*english_num+beta12*meds+beta13*night_num+beta14*vs_shock_yes+
beta15*acuity_pool2+beta16*acuity_pool3+beta17*acuity_pool4+
beta18*year14+beta19*year15+beta20*year16+beta21*year17 + b;

    /* Log-Likelihood for Normal distribution (Residual variance sigmae^2) */
    dens = -0.5*log(3.14159265358) - log(sigmae)-0.5*(resp-mean)**2/(sigmae**2);
    ll = dens;
end;

if dist = "Binary" then do;
    /* Corresponds to Eq 5: Logistic submodel */
    eta = alpha0 +
alpha1*age+alpha2*sex_num+alpha3*nonwhite_num+alpha4*hosp_Alta+alpha5*hosp_LDS+
alpha6*hosp_Riverton+alpha7*arrival_EMS+alpha8*elix_score_vw+alpha9*source_other+
alpha10*source_Urinary+alpha11*english_num+alpha12*meds+alpha13*night_num+alpha14*vs_shock_ye
s+
alpha15*acuity_pool2+alpha16*acuity_pool3+alpha17*acuity_pool4+alpha18*year14+
alpha19*year15+alpha20*year16+alpha21*year17+ lambda*b;

    /* Inverse Logit link */
    p = exp(eta)/(1+exp(eta));

    /* Log-Likelihood for Bernoulli distribution */
    ll = resp*log(p) + (1-resp)*log(1-p);
end;

model resp ~ general(ll);
/* Corresponds to Eq 2&3: Random effect b ~ normal(0, sigmab^2) subject=physician ID */
random b ~ normal(0,sigmab*sigmab) subject=attg_id;
run;
```

Qualitative data analysis

Interview transcripts were loaded into Dedoose (Sociocultural Research Associates LLC, Los Angeles, CA) for content analysis and coding. Formal notes were not taken during interview and interview subjects did not review or edit transcripts. Thematic saturation was evaluated qualitatively by consensus. A combination of inductive and deductive methods were used to analyze the transcripts from the interviews. The initial layer of coding was focused closely on the words of the participants. Clinical knowledge, existing guidelines, and data from pilot interviews were applied by a clinician investigator (IDP) and qualitative researcher (JB) to build a preliminary codebook. Using this codebook, all interviews were independently coded by two qualitative researchers. Discrepancies were reviewed by a third reviewer (JB). This reflexive process is consistent with methods to develop a thematic analysis,[19,20] however we did not produce a coding tree. In this study, we focused on preselected specific code groups related to clinical decision making, care coordination, and sepsis care protocols. The choice of these codes was informed by the threshold model of decision making under uncertainty,[21] fuzzy trace theory,[22,23] and the dual-process model of clinical decision making.[24] We reviewed all data tagged with the selected codes and chose exemplar quotes that provided context to antimicrobial initiation behavior. After illustrative codes and quotes were selected, we then revealed physician assignments to the faster or slower quartile for time to antimicrobials and, in an iterative process, discussed and synthesized interpretation of the pre-selected quotes by code.

© 2026 Peltan ID et al. *JAMA Network Open*.

eAppendix 2. Physician Interview Script

DETAIN Sepsis QUALITATIVE

Determinants of Early Treatment & Antibiotic Initiation for New Sepsis

PHYSICIAN INTERVIEW SCRIPT
Updated 18 March 2022 — Version 1.00

INTRODUCTION

Thank you for agreeing to participate in this study. You have been asked to take part in this interview because you are an ED physician who treats patients with sepsis. The interview will last about 45 minutes.

I will be audio-recording this interview.

Interviewer: *Turn on audio recorder.*

This is an interview with DETAIN subject ______[SUBJECT ID#]____.

Please speak loudly and clearly so that your comments can be captured on the recording. These recordings will be transcribed so that we can review it later. Any information that could identify you personally will not be shared with anyone outside the research team or in any publications or reports.

We want to understand your experience and decision-making in diagnosing and treating patients with possible sepsis. You do not have to answer any questions that make you feel uncomfortable or that you do not want to answer. You can end the interview at any time. We are interested in your thoughts and opinions. Some questions may sound a bit repetitive as we probe to gain additional insight. There are no right or wrong answers.

© 2026 Peltan ID et al. *JAMA Network Open*.

DEMOGRAPHICS

Interviewer: "Before we begin, I have a couple of quick background questions."

1. **How many years have your worked in an Intermountain Healthcare ED?**
2. **What race do you consider yourself?**
3. **Do you consider yourself Hispanic or Latino/a?**
4. **How old are you?**
5. **At the end of the study, our participants will all get a fleece jacket. What size do you want (they'll be unisex sizes)?**

GENERAL EXPERIENCE OF RECOGNIZING SEPSIS

Interviewer: "Now I'd like to learn more about what it's like to care for patients with sepsis in the emergency department."

***Interviewer**:* *Prompt/probe repeatedly as needed to obtain additional information. Potential prompts include:*

- *Tell me more*
- *What do you mean when you say ______?*
- *What else?*
- *Why?*

6. **When you hear the term "sepsis," what does it mean to you?**
7. **What does sepsis look like in your patients?**

   Optional probes:
   7A. How do you tell the difference between "sepsis" and non-infectious conditions that can mimic sepsis?
   7B. How do you tell the difference between simple infection and sepsis?

8. **How do you decide how sick a patient is?**
9. **What are some of the more challenging aspects of evaluating [emphasis] patients with possible [emphasis] sepsis patients in the ED?**

© 2026 Peltan ID et al. *JAMA Network Open*.

CASE-BASED EXPERIENCE OF RECOGNIZING SEPSIS

Interviewer: "I especially want to understand your thought process as you care for possible sepsis patients. It will be helpful if you could think of a recent case of possible sepsis that was challenging. I'll then walk you through your thought processes during the case. [PAUSE] Let me know when you have a case in mind … Ready?"

Interviewer: *Use info from questions #6-9 to seed probes for case-based questions. Can encourage subject to discuss thought process over case details.*

10. Did you start any testing or treatment before seeing the patient in person?

11. Why? / Why not?

12. About how long had the patient been in the ED before you evaluated them?

13. What influenced the timing of your initial evaluation?

Optional probes:
13A. Was this typical?
13B. Why?

Interviewer: *For questions #14-20, skip question if topic adequately addressed in response to prior questions.*

14. What did you feel like you needed to know to make a diagnosis?

15. Walk me through your thought process.

16. What did you do next?

17. Why?

Interviewer: *Repeat questions #16-17 as needed.*

Optional probes:
17A. What diagnostic or lab tests did you order?
17B. Why did you choose those tests and not others?
17E. What were you thinking once you received the results?

18. How did you decide how sick this patient was?

19. How likely was infection in your mind?

20. How did you decide that?

© 2026 Peltan ID et al. *JAMA Network Open*.

CASE-BASED EXPERIENCE OF TREATING SEPSIS

21. What treatments did you decide on?

22. Why?

Optional probes:
22A. Why did/didn't you give antibiotics?

Interviewer: *Read below prompt **ONLY** if case above did not receive antibiotics.*

Interviewer: "Now I'd like you to think of a patient who you diagnosed with sepsis and treated with antibiotics."

23. Once you decided to give antibiotics for this patient, what actions did you take? (e.g. order entry, communication to RN, discussion with pharmacist, shared urgency, IV access)

24. Why?

Optional probes:
24A. What did you do next?
24B. Why?

25. What influenced the timing of antibiotics for this patient?

Optional probes:
25A. What patient factors affected timing?
25B. What factors unrelated to the patient affected antibiotic timing?

© 2026 Peltan ID et al. *JAMA Network Open*.

GENERAL EXPERIENCE OF TREATING SEPSIS

Interviewer: "Now I'd like to talk a little more about treatment initiation for patients with sepsis in general."

26. When it comes to treating sepsis, what do you think is most important?

27. Why?

28. What influences your decisions regarding <u>if</u> [emphasis] a patient with suspected infection gets antibiotics?

29. Now I've heard you say ______. What else influences the <u>timing</u> [emphasis] of antibiotics once you've decided to prescribe them?

<u>Optional probes</u>:
29A. What <u>patient</u> [emphasis] factors affect antibiotic timing?
29B. What factors <u>unrelated</u> [emphasis] to the patient affect antibiotic timing?

30. Now I've heard you say ______. What other steps do you take to get antibiotics started once you've decided to prescribe them?

31. Focusing now just on <u>treatment</u> [emphasis], what are the challenging aspects of sepsis care in the ED?

© 2026 Peltan ID et al. *JAMA Network Open*.

SEPSIS BUNDLES, METRICS, AND QI

Interviewer: "We're just about done."

38. How do you feel about the use of protocols and care bundles for sepsis management?

39. What do you think about the measurement of sepsis care and outcomes?

Optional probes:
39A. How do your feelings differ depending on whether sepsis metrics and protocolized care are used for public reporting versus pay-for-performance versus quality improvement?
39B. Why?

Interviewer: *Skip question #40 if short on time.*

40. Do you have any suggestions for improving evaluation and care of possible sepsis in the ED?

CONCLUDING QUESTION

Interviewer: "Thank you so much for your time today."

41. Is there anything else you would like to tell me about treating patients with sepsis or possible sepsis in the ED?

© 2026 Peltan ID et al. *JAMA Network Open*.

STANDARDIZED PATIENT CASE

Interviewer: "Next, we're going to have you review a patient case and ask a few questions about this case."

Interviewer: *Provide or show standardized case worksheet to subject.*

Interviewer: "Please review this patient case and let me know when you're ready to continue."

32. On a scale of 0 to 10, with 10 being 100% certain, what is the probability of infection?

33. Tell me why?

Optional probes:
33A. What data points were most important?
33B. Why?

34. On a scale of 0 to 10, with 10 representing a patient in extremis, what is the patient's severity of illness?

35. Tell me why?

Optional probes:
35A. What data points were most important?
35B. Why?

36. Based on the available data, would you give this patient antibiotics?

37. Tell me why.

© 2026 Peltan ID et al. *JAMA Network Open*.

eAppendix 3. Antibiotic Spectrum Scores

Spectrum scores were assigned for each unique antibacterial antibiotic that patients received between ED arrival and discharge. Scores adapted from Stenehjem *et al.*[25] had integer values from 1 to 5, with one indicating the narrowest spectrum and five the broadest. Antiviral and antifungal medications were not assigned spectrum values.

	Antibiotic spectrum score
Amikacin	4
Ampicillin	1
Ampicillin/sulbactam	3
Azithromycin	2
Aztreonam	4
Cefazolin	1
Cefepime	4
Cefotaxime	3
Cefoxitin	2
Ceftaroline	4
Ceftazidime	4
Ceftazidime/avibactam	5
Ceftolozane/tazobactam	5
Ceftriaxone	3
Cefuroxime	2
Ciprofloxacin	4
Clindamycin	2
Colistin	5
Dalbavancin	4
Daptomycin	5
Doxycycline	2
Ertapenem	4
Erythromycin	2
Fidaxomicin	1
Gentamicin	4
Imipenem/cilastin	5
Levofloxacin	4
Linezolid	5
Meropenem	5
Metronidazole	1
Moxifloxacin	4
Nafcillin	1
Penicillin	1
Piperacillin/taxobactam	5
Rifampin	1
Tigecycline	5
Tobramycin	4
Trimethoprim/sulfamethoxazole	2
Vancomycin (IV)	4
Vancomycin (PO)	1

© 2026 Peltan ID et al. *JAMA Network Open*.

eTable 1. Patient Characteristics by Physician-Estimated Door-to-Antimicrobial Time Quartile

	Quartile 1 (fastest door-to-antimicrobial time, N=2,381)	Quartile 2 (N=2,078)	Quartile 3 (N=2,428)	Quartile 4 (slowest door-to-antimicrobial time, N=2,293)
Age, median (IQR), years	63 (48-76)	63 (50-75)	62 (48-75)	62 (47-76)
Sex at birth, n (%)				
Female	1194 (50.1%)	1029 (49.5%)	1232 (50.7%)	1180 (51.5%)
Male	1187 (49.9%)	1049 (50.5%)	1196 (49.3%)	1113 (48.5%)
English preferred language, n (%)	2234 (93.8%)	1965 (94.6%)	2296 (94.6%)	2184 (95.3%)
Hispanic/Latino or race other than White, n (%)[a]	404 (17.0%)	348 (16.7%)	420 (17.3%)	338 (14.7%)
Weighted Elixhauser comorbidity score, median (IQR)	0 (0-8)	0 (0-9)	0 (0-2)	0 (0-6)
Brought to ED by ambulance, n (%)	731 (30.7%)	657 (31.6%)	661 (27.2%)	697 (30.4%)
Nighttime ED arrival (12 am to 6:59 am), n (%)	326 (13.7%)	226 (10.9%)	189 (7.8%)	1032 (11.2%)
Triage acuity score, n (%)				
Resuscitation	30 (1.3%)	32 (1.5%)	44 (1.8%)	32 (1.4%)
Emergent	1361 (57.2%)	1203 (57.9%)	1298 (53.5%)	1215 (53.0%)
Urgent	946 (39.7%)	807 (38.8%)	1054 (43.4%)	1010 (44.0%)
Semi- or non-urgent	44 (1.8%)	36 (1.7%)	32 (1.3%)	36 (1.6%)
Mortality in ED Sepsis score, median (IQR)	6 (5-9)	6 (5-9)	6 (5-9)	6 (5-9)
SOFA score, median (IQR)	4 (3-6)	4 (3-6)	4 (3-6)	4 (3-6)
Shock present on ED arrival, n (%)	222 (9.3%)	208 (10.0%)	204 (8.4%)	191 (8.3%)
Lactate checked and >2 mmol/L, n (%)	936 (39.3%)	838 (40.3%)	892 (36.7%)	903 (39.4%)
ED-diagnosed source of infection, n (%)				
Pulmonary	953 (40.0%)	828 (39.9%)	987 (40.7%)	860 (37.5%)
Urinary	425 (17.9%)	401 (19.3%)	437 (18.0%)	454 (19.8%)
Other	1003 (42.1%)	849 (40.9%)	1004 (41.4%)	979 (42.7%)
Door-to-antimicrobial time, median (IQR), mins	133 (87-186)	147 (98-204)	165 (115-228)	182 (133-250)
Infection absent on final adjudication, n (%)	235 (9.9%)	151 (7.3%)	193 (7.9%)	199 (8.7%)
Admission to ICU from ED, n (%)	775 (32.5%)	670 (32.2%)	685 (28.2%)	716 (31.2%)
In-hospital mortality, n (%)	120 (5.4%)	111 (5.7%)	114 (5.1%)	104 (4.8%)
30-day mortality, n (%)	213 (8.9%)	185 (8.9%)	204 (8.4%)	194 (8.5%)

Values reported as median (IQR) or n (%).
Abbreviations: ED, emergency department; SOFA, Sequential Organ Failure Assessment
[a] Race other than White includes American Indian or Alaska Native, Asian, Black or African American, Native Hawaiian or Pacific Islander, and multiple or other race.

© 2026 Peltan ID et al. *JAMA Network Open*.

eTable 2. Physician Demographics by Interview Eligibility and Participation

	Overall (N=88)	Interview-eligible (quartile 1 and 4) physicians (N=44)	Physicians participating in interviews		
			Overall (N=18)	Quartile 1 (fastest) (N=9)	Quartile 4 (slowest) (N=9)
Eligible patient encounters, n	104.5 (75-129)	105.5 (77.5-138)	108 (84-108)	121 (110-147)	97 84-106)
Age, median (IQR), years[a]	39 (35-49)	39.5 (35-51)	37 (34-43)	38 (37-43)	36 (32-40)
Sex at birth, n (%)					
Female	17 (19%)	6 (14%)	4 (22%)	2 (22%)	2 (22%)
Male	71 (81%)	38 (86%)	14 (78%)	7 (78%)	7 (78%)
Years since medical school, median (IQR)[a]	11 (7-21)	11.5 (7-22.5)	9 (6-13)	11 (8-13)	8 (6-10)
Completed EM residency, n (%)	74 (84%)	36 (82%)	17 (94%)	9 (100%)	8 (89%)

Values reported as median (IQR) or n (%).
Abbreviations: EM, emergency medicine
[a] Measured at the time of physician cared for their first-eligible patient during the study period

© 2026 Peltan ID et al. *JAMA Network Open*.

eTable 3. Association of Physician Characteristics With Antimicrobial Timing

	**Change in mean door-to-antimicrobial time[a] (95% CI)**	**P value**
Years since medical school (per additional year)	-0.4 (-1.0, 0.3)	0.26
Female sex	-6.8 (-18.3, 4.8)	0.26
Completed EM residency	-7.5 (-25.1, 10.2)	0.42

Abbreviations: EM, emergency medicine

[a] Estimates for the change in mean door-to-antimicrobial time represent the coefficients and 95% confidence intervals derived by incorporating the respective physician characteristics as physician-level fixed effects in a generalized linear mixed effects model with patient door-to-antimicrobial time as the outcome, a random intercept for physician, and patient-level fixed effects for prespecified patient covariates and study hospital.

© 2026 Peltan ID et al. *JAMA Network Open*.

eTable 4. Unselected Interview Quotations Related to Preselected Codes

Faster Door-to-Antimicrobial Quartile	Slower Door-to-Antimicrobial Quartile
**Clinical Gestalt**	
#101 Some of that will be your clinical gestalt. So as you're seeing the patient in front of you, many of us that have been working in the emergency department for a number of years … you can recognize kind of the sick and not sick.	#107 Again, walking in the room and talking to the patient is my best sense. It's hard to describe … I feel like I have a sense within 30 seconds of how sick someone is.
#104 I think there is several things. I think when you walk in the room, a lot of times you can tell just by looking at a patient. Not always but sometimes you can tell just by the way they are breathing. The way they are interacting with you. The color of their skin. Then obviously vital signs play into that. If they are hypotensive or really tachycardic, or have really high fever, or I guess conversely a really low temperature. I think all of those things, you are kind of looking at all of those things together.	#116 I can't tell you I have a great algorithm, I have enough experience that I trust my gut to say, OK. That person is sick. Let's give him antibiotics now. That person is probably not sick. Let's see how they do. And I'm sure I'm wrong some of the time, but I try to just trust my instincts and then – but also second guessing. Like when that patient wasn't getting better the other night that I thought would get better, it was, OK, clearly, you're wrong about this one. It's time to be more aggressive. And so yeah, that's my general approach. I wish I could put a nice checklist for you on it with a nice outline, but it's a little less precise than that.
#113 I think experienced clinicians do develop and ability to walk into a room and just have a gestalt that this patient is sick. I think that is one times one of the traps is everybody can get mislead. And so those patients who you walk into a room and your gestalt is that they're sick they're the easy one. Your decision has been made and you put the train on the track so to speak and you're not going to take it off those tracks until the patient convinces you kind of empirically that they're not sick. The ones that are difficult are when that sense, when that sort of Spidey sense doesn't tingle. So there are a fair number of patients who end up being sick who it's only a few only sometime after when it becomes apparent. So you have this additional sense, sick or not sick but the key is to not turn off early on those who you don't have that immediate sick sense.	#117 Sometimes it's a guess as to what their primary source is. Other times it's obvious if they have flank pain and kidney pain and pyuria, then it's most likely the urosepsis and you probably just get by with ceftriaxone. Give that fairly quickly. Other times, it doesn't declare itself. … Sometimes you cannot. So, you just do your best and then err on the side of treating sepsis. Then so you could be wrong and treat sepsis, but you don't want to be wrong and miss sepsis. So, that's my approach.
#118 I would say it's, from the outset, it's really their- A gestalt of their clinical appearance, what the person looks like in bed. Are they looking sick? A person who looks sick often is going to be sick. Maybe their diaphoretic, or they're pale, or most common- The most concerning thing, maybe they are newly confused in association with SIRS type criteria. If they have any confusion associated with it, that to me suggests that they are going to become- Have a potential to become much more ill.	#120 I think definitely that that ends up being based a bit more on history and the physical exam. I think, initially, I'm always not ruling out sepsis as a possibility, if we have somebody come through triage, say they don't have a fever, but they meet other serious criteria. When later as you get initial labs, and we have the benefit of being able to get fairly rapid labs, or having concerning findings there, I'm always keeping sepsis on the differential. And if I have a fairly high clinical suspicion, based on what the patient has told me, historically, then a lot of times, I will continue to work down the sepsis algorithm, until there's something else that may come up as a more probable explanation for say, tachycardia or an elevated heart rate.

© 2026 Peltan ID et al. *JAMA Network Open*.

Faster Door-to-Antimicrobial Quartile	Slower Door-to-Antimicrobial Quartile
**Multidisciplinary Care Coordination**	
#101 Sometimes the initial workup might be normal, and then it's a matter of recognizing, reassessing, making – again, making sure we have repeat vital signs because – and then, and then not disconnecting my thoughts remote. A patient doesn't have sepsis, their workup's negative. Keeping my concern about that potential sepsis patient in my mind, and keeping it in the nurses mind to say, "Hey. Even though things look good, please let me know if anything changes." And I think that's sometimes where we miss more – the more subtle cases where the patients that may develop as they're being seen in the ED, over a number of hours. … So really when it's busy, it's rallying the team to, again, using our nurses to recognize sepsis in a similar way that I recognize sepsis. Being able to understand those sick presentations, the subtle presentations, and then really understanding when that patient can't sit up in the waiting room for two to three hours waiting for a provider.	#103 We are one of the specialties that treats before we necessarily have all the information. We have to. But delays come all over the place. Once I put antibiotics in, sometimes the nurse is busy somewhere else. Sometimes we have to get it from central. It's not actually in our Pyxis and that can take some time. But once I put the order in, the delays really are on the nursing side. … Well, you order everything at once. You see the patient. You come back to your desk. You order shotgun everything at once and then you are at the mercy of the Phleb on whether the labs come back soon. The nurse on how quickly she can get the viral swab off. The x-ray tech on how soon they can get to x-rays. I just take the information as I get it. If someone is really sick then I'll go find the nurse myself and say hey, this patient needs our attention.
#106 Communication can be a barrier, even with our Voceras. And this is again, harder in the patients that are not as sick. We do a fantastic job with the septic shock patients that you recognize that they're in septic shock. Because everyone's at the bedside, and we have direct communication. But in patients that have cold sepsis or are just a little SARS, as we call it, and you're not sure. Sometimes, like you put in a fluid order, and you assume it happens, but then it doesn't happen or takes a long time. Nurses will occasionally stop fluid orders whether I really want because they're worried we're going to volume overload the patient. So, sometimes they're right about that and make good calls. … It's typically my practice to Vocera the nurse and say, I've put in some- particularly if I've ordered cultures at the same time as antibiotics, I will routinely Vocera the nurse, so that those don't happen in the wrong order. And in- and again, in this case, I don't think the patient was decompensating that I need bedside support. But in sicker patients, then you normally Vocera our pharmacy team and the nurse, and we all kind of huddle outside the room and talk about the plan at one time.	#107 Like I said, sepsis can be subtle. And it's more than one exalted mind identifying it, it's a team sport. Bundles and protocols help empower each member of the team to impact outcomes. … There's good communication between the nurse and myself are factors outside of our control for how busy the ED was, I couldn't have seen the patient any earlier. … Again, empowering nurses and techs to engage in the likelihood of sepsis discussion and initiate risk stratification and treatment measures.

© 2026 Peltan ID et al. *JAMA Network Open*.

Faster Door-to-Antimicrobial Quartile	Slower Door-to-Antimicrobial Quartile
#109 Having enough staff and knowledgeable staff who know what needs to happen especially when we, as physicians are usually multitasking over more patients than the treating nurses. That's become more challenging. Unfortunately, because of staffing shortages and the fact that most of the nurses in the ER right now have less than a year of experience, period, 75% of the nurses, I can't count on them to come get me with abnormal vital signs or abnormal labs or a patient who's looking crummy because they don't even know what crummy looks like. I would say that it's become even more difficult for us as a team to identify early sepsis because of those concerns.<br>Well, I usually am in the room, and I ask the phlebotomist to order to draw the blood cultures while I'm looking at the patient, but I haven't gotten a history even yet. When somebody's that sick, it becomes a – if I don't do it while the phlebotomist is in there putting in the line, then there's going to be a delay to getting blood cultures because the phlebotomist is going to be in another room putting a line in somebody else when I order the blood culture. That can be like 30 to 45 minutes, which is time that we haven't given antibiotics to the patient. If I'm able to see the patient, especially the sick ones who show up right away, and we've sort of tried to train the nurses that if you see somebody who looks sick or unstable, just let us know because then we can come to the room and layout kind of what our plan is going to be, a plan of attack in order of operations so that then everybody is on board with what needs to happen at each step of the way.	#114 I think sometimes if it's not standard antibiotic that we have just in the Pyxis in the ED, sometimes there's a delay, just it takes forever for pharmacy to bring the antibiotic up. And so you're like, hey, I put this order in half an hour ago, how come you haven't been given. And the nurse is like, I have- your pharmacy hasn't brought it up yet. So I think that's a potential delay. If maybe the patient was mildly ill initially and so I kind of didn't draw blood cultures and now I ordered blood cultures and antibiotics at the same time, there's a delay because they're starting a second line drawing blood cultures. And so it takes an extra half an hour where, again, we didn't kind of go full court press in the beginning. So those are two maybe additional things to think about. … Getting that 89-year-old that you've been waiting for three hours on a urine and you're pretty darn sure that that patient is septic, but you like to wait to get antibiotics until you have that urine collected so you can get a good culture. And yet nursing staff is too busy and doesn't prioritize that. That goes by the wayside. I think sometimes it's my busyness where I'm carrying 13 patients at one time, and I know as soon as that patient walked in the door, I know they're sick, but I also am just trying to keep up with the way the patient's coming in… So, it's changing picture, limited information, poor historian, busy doctor, busy nurse, all of those things can add up to delays in treating sepsis … Most of the time, the nurse doesn't come to me and say, hey, I administered new antibiotics. The best way that I can tell that they were given is, I put an order in and once that- there's a place on my electronic, EMR that it will show up as order completed. And occasionally- again, I'm super busy sometimes, so I can't always micromanage that, but occasionally if I notice, hey, that still hasn't been done, I'll try to'touch base with the nurse and sa', hey, did you have a chance to give 'ntibiotics. And sometimes it's a'line issue or sometimes they were just busy and had something else on.
#111 I usually always tell the nurse. On a patient like this, I probably have the nurse in the room anyway because we are doing a lot of things and trying to figure things out. I told the nurse, here's what I'm going to order. Let's administer this antibiotics after you get the blood cultures. Then I leave the room and try to remember to go right away. On him I did because I had a lot of orders to put in. But right away over to the computer and enter the orders, and make sure I enter the antibiotic while I'm at it.	#115 So, the way it happened is she, of course, checked into labor and delivery first, then if they have a patient that they don't feel comfortable managing, they will call us. So, we got a heads-up that they were sending her down. They called down to say that it was because of a fever, that she was 30 weeks pregnant, and they sent her down to us, so we had at least received that heads up and they wheeled her down to the department. And so, I already knew through our charge nurse that she was coming, and that's part of why I was in the room so quickly as well.

© 2026 Peltan ID et al. *JAMA Network Open*.

Faster Door-to-Antimicrobial Quartile	Slower Door-to-Antimicrobial Quartile
#113 It also is really highly dependent on nursing both nursing workflow and kind of individual nurses. This patient has a nurse who's an experienced nurse but who moves a little bit slowly and so everything doesn't get done at once. The kind of the thing that, I think the nurse prioritized appropriately and this patient got early antibiotics. He was really, really slow to collect a urine sample and COVID testing and actually a urinary tract infection ended up being his likely source of – and so it kind of depends on nursing prioritization and nursing experience and everything like that. … The best critical care nurses whether it be in the emergency department or in any venue are ones who are comfortable multitasking and prioritizing. So there's no question that the very best nurses seem to make everything happen simultaneously.	#120 Yes, I think the big thing, obviously, is getting them ordered. But definitely, if I've decided to do antibiotics, I always communicate directly with the nurse, like I said, are experienced self-motivated nurses, I probably won't go beyond that. Because I know that they will themselves talk to the pharmacist and call the inpatient pharmacy and get things going. I'll ask the nurse to kind of go in there early, try to get things done early.<br>And so I'll usually involve the pharmacists for them to say, I need antibiotics, can you make sure they're coming from pharmacy and that you deliver into the room and kind of prompt the nurse to get them started and here's why. But with this nurse, I did not do that. And she got things started, I think probably within the next 15 minutes after I talked to her.

© 2026 Peltan ID et al. *JAMA Network Open*.

Faster Door-to-Antimicrobial Quartile	Slower Door-to-Antimicrobial Quartile
#118 I might talk to the nurse and just say hey, I ordered antibiotics for this patient. Will you look for them when they come from the pharmacy? They need to be infused. But usually if it's sort of my gestalt is that the timing does not matter all that much as far as if they get them an hour after they arrive to the emergency department versus an hour and 20 minutes after they arrive that the outcome is not appreciably different. But if it clearly looks like they need them, they're going to need them regardless of their labs, then I might just say talk to the nurse. This is what we need to do. Usually if they understand the reasoning behind, then they understand that you want them sooner rather than later. Usually and in this particular case the same. But usually what happens is that if a patient, again, is recognized as having symptoms that could be of a serious nature, whether it's infection or not, in her case it was not highly suspicious for infection just passing out. It's still a concerning symptom to have at 80 something years old. They get put in a room, get put in a monitor and an IV gets established even without my say so. Someone is usually in there before I'm in there doing their job and then they wait for me to put the actual orders in the computer to send the blood that they've already taken out of the patient. Usually IV access is established in most patients sort of as an automatic thing no matter what emergency room you're at. And I would say, again, they usually will have a more experienced person out in front of the ER because it does take an experienced nurse to recognize sick from not sick. You might get a nurse who doesn't quite get the under- Understand that a 20-year-old female with a fever of 39 and a heart rate of 115 is not going to likely be as sick as a 70-year-old female with a heart rate of 105 and a temperature of 38.1. The vitals look better on the older person but the older person has a higher likelihood for decompensation due to so many things but primarily due to lack of ability to continue to compensate at that rate. I would say in most ERs, a triage nurse is commonly going to be a more experienced nurse, thought that may not always be the case, I think that's commonly the case. And so, they have to recognize it first to say, this person is sick, send them back.	
**Protocols and Care Bundles**	
#101 I feel that they improve my ability to make timely decisions. It allows me to have all the appropriate orderables, and I – on that same pathway. So I think it really helps improve the compliance with substance on my end.	#103 I think some protocols and care bundles are helpful. … We really just had a checklist and, patients, once they fell into the appropriate service criteria, they got antibiotics and admitted, and you were done. Here, they really like to get to an answer more. The protocol meant that we didn't really miss people with sepsis. Here, we do have protocols and bundles and they're helpful.

© 2026 Peltan ID et al. *JAMA Network Open*.

Faster Door-to-Antimicrobial Quartile	Slower Door-to-Antimicrobial Quartile
#104 I think they have their place. I think it's in the trajectory of my career I think it's good. I'm thinking that we better care of septic patients than we used to. I mean there's much less sort of – you know I think we jump on patients quicker than we used to and so, I think in that respect it's been good. I think it annoys me a little bit because I think we kind of what I eluded to earlier is that I think sometimes with these protocols and sort of having them become dogma, I think sometimes the more nuanced cases, like that last case you showed me can get, it can kind of force us to turn our brains off a little bit, which I don't like. … So, I guess I'm a little mixed on it. But I think, I don't know my feeling is the sepsis thing has probably been overall a good thing, because I do think people probably get better care than they used to.	#107 I like them. *#And why?*<br>I've seen they improve outcomes across different care spaces that might struggle. Like I said, sepsis can be subtle. And it's more than one exalted mind identifying it, it's a team sport. Bundles and protocols help empower each member of the team to impact outcomes.
#105 I like them. *#And why? Extrapolate, tell me more.*<br>Well, particularly with the EMR, there's so many places that I would have to go, to click boxes to get things done. And the sepsis bundle allows me to pull it all on the one page and click what I don't want. So it's much easier to click things off than it is to remember what to click on, I think.	#110 I like protocols that kind of decreases variability. I do not like bundles because the bundle is like a one size shoe. For a lot of different sized feet.
#106 Foreign. Can I say that in one word? I find them helpful and distracting, at the same time. I think it's helpful too, so my job is unique in that it is defined by interruptions in many ways because we're so busy in the ER. … There's like the prompts and the queues, and then there's the bundles. I think of those as quite different. I think bundles are more helpful in making sure that we don't admit important care. I think the prompts can lead to a degree of alarm fatigue, like for example, with all the viral sepsis we're seeing. Obviously, just increase the amount of distractions in the ER, which at some point decreases my clarity of thought, if I'm overly distracted. So, I don't know where the right line there is. Back to bundles. You know, everyone's complained about the bundles, is that they treat everyone one size fits all. We can't give for septic shock, for example, or lactic acid over four we can't give everyone 30 MLs per kilogram of fluid, which is part of the bundle. We'll do some patients harm, and so people get frustrated by that.	#114 Generally I'm in favor of them. I think they're good to help remind us, hey, we need to think about this, we need to think about this early. I think sometimes you- it is challenging. I think I mentioned fluid administration, there are definitely nuances that bundles don't necessarily take into account or appreciate. But I still feel like they still get us on the right track for the majority of the patients. I think it's helpful sometimes even in antibiotic selection. And I think just when there are some click boxes in there, sometimes it helps remind you, I didn't order sputum cultures on this patient, or here's something else I needed to consider. So generally I'm a fan.

© 2026 Peltan ID et al. *JAMA Network Open*.

Faster Door-to-Antimicrobial Quartile	Slower Door-to-Antimicrobial Quartile
#108 I think I'm probably middle of the road. I don't hate them. I don't love them. I don't get, but I don't, it helps in the sense of having, you consider it maybe at times when you wouldn't. I do think it's pretty nonspecific, especially when it comes to viral seasons or dealing with COVID patients for example. But so I think I'm middle of the road. I don't have strong feelings about it. I think overall, I guess I accept that the data, that early identification and treatment is compelling enough that it's, should be a focus of our care. I have some concerns when it pulls care, it pulls resources towards a patient that doesn't really need them and away from others. But I feel like we are at a reasonable place currently in how we try to flag it and pay attention to it. It doesn't strike me as overly done.	#115 How do I feel in general? I think they're good in general. I think- yes, overall I like them. I think it simplifies things a little bit when it comes to some of the treatment decisions. I'd say I'm for them. I'm for protocols in general. So, I guess overall, if you're just looking for my feelings, I'm for them.
#109 I think for the right patients, it's wonderful especially with new nursing staff because they can get used to what needs to be ordered. It also helps us not forget things. If we order a la carte and forget the lactate and miss the lactate, then that's a problem. I do like it for that. I think it also assumes that we know what the pathology is while we're ordering the tests when we're trying to order the test to figure out what the pathology is. I think it's really helpful for antibiotic choices, especially when we're at the smaller facilities where we don't have an ed pharmacist on there because then we don't have to think about what do I need to give this person? You can just click the boxes, which is great. Because especially when you're managing a small department and maybe your nurses aren't as used to taking care of sick patients, it takes one more cognitive exercise off of your list to do that.	#116 So, I am Jekyll and Hyde on this subject. I really like the sepsis. We have two bundles. I really like the sepsis workup bundle. So that's the diagnostic bundle, so that is a checklist you can go through and order the test to figure out if somebody's septic, and really put your foot on the gas to get that all done quickly. It gets the team and the nurses involved very quickly. I like that a lot. I've tried to use it exclusively any time sepsis or infection enters my brain. … The diagnostic bundle, that is super useful. I find the treatment bundle a little more challenging, and that's probably because I – my confirmation or my availability bias is remembering the cases where it wasn't clear. I think this, the treatment bundle is super easy when you walk out of a room and you're, oh, that patient has a severe skin infection, because you've seen it on exam, and you go access the skin and soft tissue sepsis treatment protocol and you just execute it. Those cases don't stick in my brain because they're straightforward …<br>But so, you have to make a decision to execute the no-source bundle, which is the big-gun antibiotics, which are not harmful at a high percentage rate but are harmful to patients. That's a lot of medication, and that I find that the treatment recommendation just to be a little more frustrating, and that's not because they're not good or evidence based. It's just because there's no certainty that they're there. You don't know if you're doing the right thing, and I don't like doing something just for the sake of doing something, but I appreciate that we probably should be. So that's why I'm a little bit split.

© 2026 Peltan ID et al. *JAMA Network Open*.

Faster Door-to-Antimicrobial Quartile	Slower Door-to-Antimicrobial Quartile
#111 Love them. I think it's great. One thing I really like about it is it's hard for me personally to stay on the most current literature of everything that I'm treating. Sepsis I know that changes a lot with we should choose this antibiotics or based on our resistances, we want to be on these antibiotics. I love to have the crutch of saying I'm going to go to the sepsis order set, and I know that, or I have faith that, whoever is putting these in here is keeping them up to date on what they think is proper and appropriate. Then I'll just go down and I'll choose what I think the most likely source is, and then it'll give me suggestions on antibiotics and I'll do that.	#117 I actually like them. We use them- We actually use them a lot.
#113 I think they're great. I think they're really helpful item.	#119 I think I like it. I think it makes it easy. … And I think if like this patient would probably fall under that category where there's enough that we would say, yes, we're worried about sepsis, let's just do the sepsis bundle and then you don't have to think very hard about what that involves. It's more of a, just a one button, do all the sepsis stuff and then it's blood cultures, by dehydration, antibiotics, and you don't have to worry too much about what you're doing, if it's just we're concerned for sepsis, let's do all the things that we typically do. So I think, overall, it's helpful, it makes it easier to cast a wide net and catch all of the potentially infected patients.
#118 I would say they are helpful and not helpful, both. They are completely distracting when the person that you have in front of you is clearly a healthy person with a viral illness or a bacterial illness, which will require minimal to no treatment. I think they're probably most helpful in those patients who it's not clear that they have an infection in the first place. Gathering a list of data that says hey, this person might have sepsis. That might direct you to ask some questions about infectious etiologies. But I think it's really kind of distracting to have a significant number of people that you see in the ER who have either simple viral or simple bacterial problems and you've got to go look at an alert to basically to check a box off so that you don't have a bad mark on your record for not making that visit to that protocol page. But I think that it is probably most helpful in the patients who aren't clearly septic. But the gathering of data suggests hey, there might be sepsis here. Because in a person who it's obvious that they have sepsis, it's obvious that you need to take care of it. In the person who's obvious that who that they have sepsis but it's a minor version of sepsis, either viral or simple bacterial, that doesn't require in my opinion jumping on something. So a protocol doesn't necessarily help me as much. It's more that middle ground of maybe I didn't think about that, or I did think about it but now I'm thinking about even more because this protocol has shown me that it looks like this patient maybe have sepsis and it might rise to a higher level in my differential of thinking about what they have.	#120 I think practicing medicine with protocols and care bundles, well, it is frustrating. I do think that it helps particularly in a busy setting as a prompt to make sure that you are paying attention to things that could get initially glossed over in particularly where things are time sensitive in terms of patient outcome and appropriate care. I think that they're very helpful that way. I do think care bundles do make it easier from a communication standpoint where you can have a cohesive plan, particularly in a big easy ER where pharmacy nurses imaging yourself texts understand that when there's this clinical picture, we need to keep in mind these things. And there's less ambiguity in terms of what direction we need to go in as a care team. And it's, I think, easier to get education for all of us to make sure that we're following outcomes and that everyone is preemptive about initiating those things. So at least in sepsis or STEMIs or everything. It's all protocol driven and care bundle driven, I think in most big ERs across the country now. So I do think that it is beneficial and helpful in that regard. I think the clinical evaluation at the bedside and using those to guide and prompt your care but not dictate your care, I think is helpful and still needs to be kept in mind because I think a lot of people will fit and do well with that. But I think you also have to take into account social considerations, ethical considerations, complicated clinical pictures in terms of applying protocols and care bundles, and that's what we're here for.

© 2026 Peltan ID et al. *JAMA Network Open*.

eFigure 1. Criteria for Retrospective Adjudication of ED Infection Presence

**Overtreatment (infection ruled out)**	**Not infected**	Infection clinically determined to be absent (i.e. alternative diagnosis made) and/or infection deemed of sufficiently low probability that patient not administered a complete antimicrobial course.
**Not overtreatment (infection not ruled out)**	**Possible**	Clinical suspicion for infection (usually indicated by a course of antimicrobial therapy if applicable) and clinical syndrome likely consistent with the infectious diagnosis (generally 1-2+ symptoms, signs, and/or laboratory/imaging findings) but alternative diagnosis considered to be as or more likely than infection and/or lack of response to appropriate therapy.
	**Probable**	Clinical syndrome consistent with the infectious diagnosis (generally 2-3+ signs, symptoms, and/or laboratory/imaging findings) and infection considered more likely than other diagnoses as the cause of the patient's syndrome or clinical response to appropriate treatment but no positive culture or positive microbiologic diagnostic test for a pathogen consistent with the infectious syndrome.
	**Definite**	Infectious syndrome consistent with the infectious diagnosis and a positive culture or positive microbiologic diagnostic test for a pathogen consistent with the infectious syndrome.

Table included in figure reprinted from Hooper GA et al. (Concordance Between Initial Presumptive and Final Adjudicated Diagnoses of Infection Among Patients Meeting Sepsis-3 Criteria in the Emergency Department. *Clin Infect Dis*. 2023;76(12):2047–2055)[6] by permission of Oxford University Press and the Infectious Disease Society of America.

© 2026 Peltan ID et al. *JAMA Network Open*.

eFigure 2. Patient and Physician Participant Inclusion-Exclusion Diagram

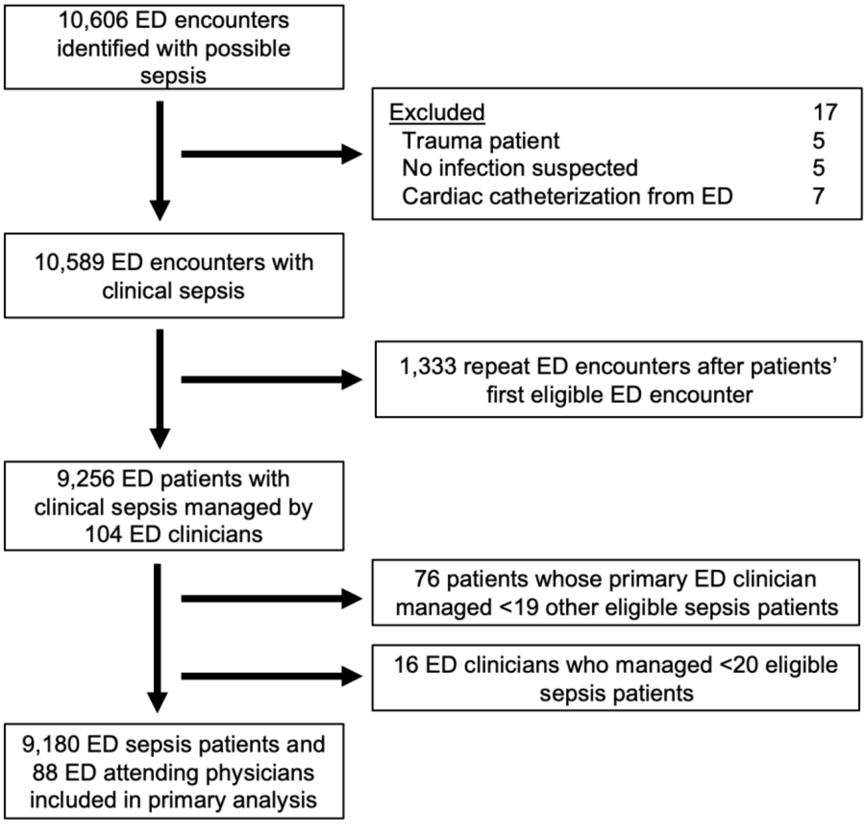

© 2026 Peltan ID et al. *JAMA Network Open*.

eReferences

1. Miller RR, 3rd, Dong L, Nelson NC, et al. Multicenter implementation of a severe sepsis and septic shock treatment bundle. *Am J Respir Crit Care Med*. 2013;188(1):77–82. doi:10.1164/rccm.201212-2199OC
2. Singer M, Deutschman CS, Seymour CW, et al. The Third International Consensus Definitions for Sepsis and Septic Shock (Sepsis-3). *JAMA*. 2016;315(8):801–10. doi:10.1001/jama.2016.0287
3. Vincent JL, Moreno R, Takala J, et al. The SOFA (Sepsis-related Organ Failure Assessment) score to describe organ dysfunction/failure. On behalf of the Working Group on Sepsis-Related Problems of the European Society of Intensive Care Medicine. *Intensive Care Med*. 1996;22(7):707–10. doi:10.1007/BF01709751
4. Peltan ID, Bledsoe JR, Oniki TA, et al. Emergency Department Crowding Is Associated With Delayed Antibiotics for Sepsis. *Ann Emerg Med*. 2019;73(4):345–355. doi:10.1016/j.annemergmed.2018.10.007
5. Peltan ID, Brown SM, Bledsoe JR, et al. ED Door-to-Antibiotic Time and Long-term Mortality in Sepsis. *Chest*. 2019;155(5):938–946. doi:10.1016/j.chest.2019.02.008
6. Hooper GA, Klippel CJ, McLean SR, et al. Concordance Between Initial Presumptive and Final Adjudicated Diagnoses of Infection Among Patients Meeting Sepsis-3 Criteria in the Emergency Department. *Clin Infect Dis*. 2023;76(12):2047–2055. doi:10.1093/cid/ciad101
7. Peltan ID, McLean SR, Murnin E, et al. Prevalence, Characteristics, and Outcomes of Emergency Department Discharge Among Patients With Sepsis. *JAMA Netw Open*. 2022;5(2):e2147882. doi:10.1001/jamanetworkopen.2021.47882
8. The Canadian Triage and Acuity Scale — Combined adult/paediatric educational program (participant's manual). *Canadian Association of Emergency Physicians*. 2013;doi:papers3://publication/uuid/D073551F-E451-4C98-8C90-775A80B92667
9. Elixhauser A, Steiner C, Harris DR, Coffey RM. Comorbidity measures for use with administrative data. *Med Care*. 1998;36(1):8–27. doi:papers3://publication/uuid/85CF3073-C759-41B1-B897-DB82D6E08D63
10. van Walraven C, Austin PC, Jennings A, Quan H, Forster AJ. A modification of the Elixhauser comorbidity measures into a point system for hospital death using administrative data. *Med Care*. 2009;47(6):626–33. doi:10.1097/MLR.0b013e31819432e5
11. Shapiro NI, Howell MD, Talmor D, Donnino M, Ngo L, Bates DW. Mortality in Emergency Department Sepsis (MEDS) score predicts 1-year mortality. *Crit Care Med*. 2007;35(1):192–8. doi:10.1097/01.CCM.0000251508.12555.3E
12. Shapiro NI, Wolfe RE, Moore RB, Smith E, Burdick E, Bates DW. Mortality in Emergency Department Sepsis (MEDS) score: a prospectively derived and validated clinical prediction rule. *Crit Care Med*. 2003;31(3):670–5. doi:10.1097/01.CCM.0000054867.01688.D1
13. Clark RE, Feldon DF, van Merriënboer JJG, Yates KA, Early S. Cognitive Task Analysis. In: Spector JM, Merrill MD, van Merriënboer JJG, Driscoll MP, eds. *Handbook of Research on Educational Communications and Technology*. 3 ed. Erlbaum/Routledge; 2008:577–593.
14. Peltan ID, Mitchell KH, Rudd KE, et al. Physician Variation in Time to Antimicrobial Treatment for Septic Patients Presenting to the Emergency Department. *Crit Care Med*. 2017;45(6):1011–1018. doi:10.1097/CCM.0000000000002436
15. Dunson DB, Chen Z, Harry J. A Bayesian approach for joint modeling of cluster size and subunit-specific outcomes. *Biometrics*. 2003;59(3):521–530. doi:papers3://publication/uuid/984F8558-EFB5-4943-A6F4-AC78F61CA2E3
16. Skrondal A, Rabe-Hesketh S. *Generalized latent variable, multilevel and panel modelling*. CRC Press; 2002.
17. Goldstein H, Carpenter J, Kenward MG, Levin KA. Multilevel models with multivariate mixed response types. *Statistical Modelling*. 2009;9(3):173–197. doi:papers3://publication/doi/10.1177/1471082X0800900301
18. Shahian DM, Normand SL, Torchiana DF, et al. Cardiac surgery report cards: comprehensive review and statistical critique. *Ann Thorac Surg*. 2001;72(6):2155–2168. doi:papers3://publication/uuid/178A389E-A70F-40A4-8D82-58E6435D2473
19. Braun V, Clarke V, Hayfield N, Terry G. Thematic Analysis. In: Liamputtong P, ed. *Handbook of Research Methods in Health Social Sciences*. Springer Singapore; 2019:843–860.

© 2026 Peltan ID et al. *JAMA Network Open*.

20. Braun V, Clarke V. Conceptual and design thinking for thematic analysis. *Qualitative Psychology*. 2022;9(1):3–26. doi:10.1037/qup0000196
21. Pauker SG, Kassirer JP. The threshold approach to clinical decision making. *N Engl J Med*. 1980;302(20):1109–17. doi:10.1056/NEJM198005153022003
22. Reyna VF. A new intuitionism: Meaning, memory, and development in Fuzzy-Trace Theory. *Judgm Decis Mak*. 2012;7(3):332–359.
23. Reyna VF. How People Make Decisions That Involve Risk: A Dual-Processes Approach. *Curr Dir Psychol Sci*. 2004;13(2):60–66. doi:10.1111/j.0963-7214.2004.00275.x
24. Croskerry P. A universal model of diagnostic reasoning. *Acad Med*. 2009;84(8):1022–8. doi:10.1097/ACM.0b013e3181ace703
25. Stenehjem E, Hersh AL, Sheng X, et al. Antibiotic Use in Small Community Hospitals. *Clin Infect Dis*. 2016;63(10):1273–1280. doi:10.1093/cid/ciw588

© 2026 Peltan ID et al. *JAMA Network Open*.
