# Supplementary material for: Physician Variation in Early Sepsis Management
Source: JAMA Netw Open. 2026 Feb 13;9(2):e2556945. doi: 10.1001/jamanetworkopen.2025.56945 (PMC12905659; doi:10.1001/jamanetworkopen.2025.56945)
Supplement: Supplement 2. — Data Sharing Statement [file jamanetwopen-e2556945-s002.pdf]

Data Sharing Statement

Peltan. Physician Variation in Early Sepsis Management. *JAMA Netw Open*. Published February 13, 2026. doi:10.1001/jamanetworkopen.2025.56945

Data
Data available: Yes

Data types: Deidentified datasets

How to access the data: Upon request from the Intermountain Health Office of Research (officeofresearch@imail.org)

When available: For use in methodologically sound research after completion of IRB review and required data use agreements
